# Using the UK reference population Avalon × Cadenza as a platform to compare breeding strategies in elite Western European bread wheat

**DOI:** 10.1007/s11032-015-0268-7

**Published:** 2015-02-05

**Authors:** Juan Ma, Luzie U. Wingen, Simon Orford, Paul Fenwick, Jiankang Wang, Simon Griffiths

**Affiliations:** 1 Institute of Crop Science, The National Key Facility for Crop Gene Resources and Genetic Improvement, and CIMMYT China, Chinese Academy of Agricultural Sciences, No. 12 Zhongguancun South Street, Beijing, 100081 China; 2 John Innes Centre, Norwich Research Park, Norwich, NR4 7UH UK; 3 Limagrain UK Limited, Rothwell, Market Rasen, Lincolnshire, LN7 6DT UK

**Keywords:** Phenotype prediction, Simulation, QTL linkage, Pleiotropy, Breeding by design

## Abstract

Wheat breeders select for qualitative and quantitative traits, the latter often detected as quantitative trait loci (QTL). It is, however, a long procedure from QTL discovery to the successful introduction of favourable alleles into new elite varieties and finally into farmers’ crops. As a proof of principle for this process, QTL for grain yield (GY), yield components, plant height (PH), ear emergence (EM), solid stem (SS) and yellow rust resistance (*Yr*) were identified in segregating UK bread wheat reference population, Avalon × Cadenza. Among the 163 detected QTL were several not reported before: 17 for GY, the major GY QTL on 2D; a major SS QTL on 3B; and *Yr6 on* 7B. Common QTL were identified on ten chromosomes, most interestingly, grain number (GN) was found to be associated with *Rht*-*D1b*; and GY and GN with a potential new allele of *Rht8*. The interaction of other QTL with GY and yield components was discussed in the context of designing a UK breeding target genotype. Desirable characteristics would be: similar PH and EM to Avalon; *Rht*-*D1b* and *Vrn*-*A1b* alleles; high TGW and GN; long and wide grains; a large root system, resistance to diseases; and maximum GY. The potential of the identified QTL maximising transgressive segregation to produce a high-yielding and resilient genotype was demonstrated by simulation. Moreover, simulating breeding strategies with F_2_ enrichment revealed that the F_2_–DH procedure was superior to the RIL and the modified SSD procedure to achieve that genotype. The proposed strategies of parent selection and breeding methodology can be used as guidance for marker-assisted wheat breeding.

**Electronic supplementary material:**

The online version of this article (doi:10.1007/s11032-015-0268-7) contains supplementary material, which is available to authorized users.

## Introduction

Wheat breeders select simultaneously for qualitative traits controlled by one or a small number of major genes and quantitative traits controlled by multiple genes which may be detected as quantitative trait loci (QTL). There are many complex choices to be made, from the selection of the best parents for a cross through to selection strategies that result in combining multiple desired alleles into a single target genotype, all at minimum cost to the breeding programme. It is therefore a lengthy procedure from gene discovery to superior varieties in farmers’ fields. Grain yield (GY) improvement is the main objective in wheat breeding programmes. On the whole, GY improvement is achieved by selecting and crossing high yielding lines. In the UK, an increase of yields from 3 t/ha in 1950s to 7.4 t/ha in 2013 have been achieved, largely through this approach (http://archive.defra.gov.uk/foodfarm/landmanage/climate/documents/climate-ag.pdf; https://www.gov.uk/government/publications/agriculture-in-the-united-kingdom-2013). However, the on-farm GY is below the potential shown under perfect conditions, where GY of more than 10 t/ha are possible. In order to create more resilient varieties, which perform better under on-farm conditions, more diverse breeding targets are needed, which will need more genetic input in order to achieve them. The UK reference population Avalon × Cadenza was developed as a tool for the genetic improvement of wheat, as part of the Wheat Genetic Improvement Network (WGIN) (http://www.wgin.org.uk/). The Avalon × Cadenza segregating population represents a broad spectrum of elite UK winter germplasm produced in different UK wheat breeding programmes. The population has been widely used to assess multiple traits via QTL analysis such as: grain size and shape (Gegas et al. 2010), plant height (PH) (Griffiths et al. 2012), flowering time (Griffiths et al. 2009), root system (Bai et al. 2013) and mosaic disease (Bass et al. 2006). The usefulness of this population is enhanced by the continuous improvement of the genetic map, which has now developed into a very high density map (Allen et al. 2011; Wang et al. 2014).

GY is a complex trait and is determined by yield component traits. At the top level, GY is the product of thousand grain weight (TGW) and grain number (GN). On the next level, TGW is composed of grain size and grain width (GRW). Large grain size has been an important trait selected during domestication and wheat breeding (Pozzi et al. 2004), but it is GN that is most strongly associated with genetic gains in GY (Peltonen-Sainio et al. 2007). GN is determined by GN per ear and ear per square metre. There is a trade-off between the increase in GN and the reduction in TGW (Acreche and Slafer 2006). However, Sinclair and Jamieson (2006) proposed that it is not GN that determines GY in wheat, but that GN is the consequence of GY. Fischer (2008) considered that TGW and GN are linked only by the fact that GN determines post-anthesis sink size, with possible negative consequences for TGW if source is scarce. Furthermore, the potential TGW can be influenced by events as early as 1 week before anthesis but also by later events, as grains can abort after fertilisation (Duggan and Fowler 2006). This leaves possibilities for simultaneous adjustment of GN and grain size to future conditions as signalled by conditions around flowering (Fischer 2008). Even so, in order to increase GY potential, while avoiding the negative relationship between TGW and GN, it is useful to study trade-offs between yield components through QTL analysis. Marker-assisted selection (MAS) for improvement both in TGW and GN might provide a means to maximise both traits.

GRW and grain length (GRL) are the major components of TGW (Breseghello and Sorrells 2006; Gegas et al. 2010; Okamoto et al. 2013). In particular, Calderini et al. (2013) proposed that GRL is a key driver of TGW determination. Therefore, increasing either or both traits could be a wheat breeding target, though so far GRL seems to be more responsive trait.

To achieve high yields in the farmers’ fields, more than just high yield potential, which is determined by the yield component traits, is needed. Adaptive plant architecture, such as ideal PH would be another important component. Moreover, flowering time variation allows wheat cultivars to be adapted to target environments, and thus to perform more productively. Again, as with the yield components, trade-offs between traits may hinder the efforts to breed ideal genotypes. For example, common QTL for PH and ear emergence (EM) link early EM with increased height in the Avalon × Cadenza population (Griffiths et al. 2012). To produce genotypes with early EM but medium height, potential trade-offs at these common loci will need to be carefully assessed. High yield in UK is also dependent on early autumn drilling, so a successful UK wheat variety needs to be winter type.

Wheat varieties with greater resilience and productivity under water-limited growth conditions are also highly desirable. The plant architecture trait solid stem (SS) has positive phenotypic correlation with GY resilience under water stress (Saint Pierre et al. 2010) and is also known for conferring resistance to wheat stem sawfly (Houshmand et al. 2003, 2007) and lodging tolerance (Berry et al. 2007). Breeding SS varieties could thus effectively reduce yield losses under stressful environmental conditions.

Conscious selection for root system architecture has not been a prominent target in winter wheat breeding programmes. However, larger root systems contribute to increasing soil exploration and underground water and nutrient acquisition, as well as facilitating plant adaptation to water-limited environments where the wheat plant relies largely on seasonal rainfall (Palta et al. 2011). A number of seedling root trait QTL in Avalon × Cadenza were reported by Bai et al. (2013). Common QTL for roots and PH were found on 2D and 4D, the direction of additive effects was different at both loci, linking height-increasing effects to reduced root surface or length (Bai et al. 2013). Moreover, a connection between the known semi-dwarfing genes *Rht*-*B1*, *Rht*-*D1*, *Rht*-*8* and *Rht12* and root proliferation has been found (Bai et al. 2013). The trade-offs between selection for alleles for optimal medium PH and their possible adverse effect on the size of the root system need to be taken care of. The dense fibrous root system is a difficult trait to be selected for directly by breeders (Nagel et al. 2009). Therefore, MAS for root trait QTL promises to help breeders select these traits more easily.

GY losses due to plant diseases are an increasing problem in many crops including wheat. Disease resistance genes may play an important part in GY protection, but this field is too large to fully cover it here. With the focus on Avalon × Cadenza only, the resistance loci identified to the best of our knowledge in this population are mentioned. Yellow rust, caused by *Puccinia striiformis f.* sp. *tritici*, is one of the most damaging diseases for Northern European wheat. Breeding resistant cultivars is an economical and environmentally acceptable approach to control yellow rust. The identification of resistance genes and closely linked molecular makers for MAS is therefore of great interest. Mosaic disease, caused by soil-borne cereal mosaic virus (SBCMV), is another serious constraint to winter wheat production in Europe (Clover et al. 2001). Cadenza carries the resistance allele at locus, *Sbm1*-*5D*, and available markers for this disease have been identified (Bass et al. 2006; Perovic et al. 2009), which could be used to select the favourable allele via MAS.

Up to now, a large number of QTL for GY, yield component traits such as GRW, GRL and TGW, and disease resistance in wheat have been published (Zhang et al. 2010; Rustgi et al. 2013; Bansal et al. 2014). However, the identified QTL have not been routinely assessed and included in breeding programmes. More specifically, a detailed understanding is needed on how molecular markers can be best utilised to improve a complex trait. With the increasing availability of molecular markers and the increased affordability of genotyping, more and more breeding researches are dedicated to the exploitation of those favourable alleles. However, molecular breeding now faces the problem of integrating QTL findings from different mapping populations. The QTL method is not able to account explicitly for segregation of different allelic combinations among different parents and for the context dependency of QTL effects. Inconsistency of QTL findings caused by the context dependency of QTL effects is due to QTL-by-genetic background interaction and QTL-by-environment interaction (QEI). The context-dependency issues of QTL effects lead to questions about the generalizability of QTL findings. This means that the usefulness for MAS may be restricted to cases where target genotype had been determined separately for each population and the QTL detection experiments had sampled representative environments (Sebastian et al. 2010). Fortunately, molecular marker technology has become cheaper and faster. Gains per breeding cycle are thus not necessarily greater with MAS than with phenotypic selection, but the use of molecular markers can increase the genetic gain per year and per unit cost (Bernardo 2008). Genetic gain per unit cost and time rather than gain per cycle should be considered when MAS is applied in plant breeding. Examples of QTL that have been successfully used in wheat breeding by MAS include resistance and grain quality alleles (Anderson et al. 2008; Tyagi et al. 2014). Considering the cost and low accuracy associated with phenotypic selection, MAS has potential to improve breeding for complex traits, even if limited to a specific genetic and environmental context (Sebastian et al. 2010).

Using QTL information for traits of interest, including flanking markers, allelic variation and additive effect can enable breeders to design a superior genotype combining all favourable alleles at all selected loci (Peleman and van der Voort 2003). This so-called breeding by design (Peleman and van der Voort 2003; Wang et al. 2007a, 2011) has the benefit that traits do not need to be expressed for selection. MAS can help to accurately select all loci of interest, which will be particularly useful for traits that are difficult to select. Simulation software can provide a new way to evaluate new genotypes in silico, using multiple alleles, pleiotropy and epistasis models. Furthermore, it promises to be particularly helpful to determine the optimal breeding methods to obtain the target genotype, thus saving breeding time as well as field trial costs.

Here, we use the UK reference population Avalon × Cadenza as proof of concept for achieving systematic genetic gain in elite UK germplasm. Our objectives were (1) to use a high-density map to identify QTL for GY, GN, SS and yellow rust resistance (*Yr*) gene *Yr6*, and to remap TGW, GRW, GRL, PH and EM QTL by multi-environment analysis and re-identify *Yr7*; (2) to study trade-offs between GY with other traits; (3) to predict the performance of GY, PH and EM for some perfect genotypes generated by simulation under different environments; (4) to design a superior genotype comprising all or if not most favourable alleles based on QTL identified here and published QTL and genes (root traits, *Sbm1* and *Vrn*-*A1b*), and to compare the efficiency of three breeding procedures involving MAS in terms of genetic gain and number of target lines retained from one breeding cycle through simulation.

## Materials and methods

### Plant materials

The UK reference segregating population, consisting of 201 doubled haploid (DH) wheat genotypes derived from an Avalon × Cadenza cross, developed by Clare Ellerbrook, Liz Sayers, and the late Tony Worland (John Innes Centre), was used in this study. The population and parents Avalon and Cadenza were grown in Church Farm, Norwich, UK, from 2005 to 2008 (Griffiths et al. 2009, 2012) and all phenotypic scores, except yellow rust scores, were taken on these trials. PH and EM measurements were described by Griffiths et al. (2009, 2012). The observed values of EM were adjusted to percentage of the mean for better comparison between years. GY was recorded per plot in all years. Morphometric measurements for TGW, GRW and GRL were conducted in 2007 and 2008 (Gegas et al. 2010). GN was calculated from GY and TGW. SS was determined in 2005 and 2006 as percentage of fill of the total stem cross section from measurements of width and wall thickness 11 cm below the collar, using digital callipers. Ten stems were sampled in 2005 and five in 2006, choosing plants randomly from one of the replicate plots. Two sets of 10-day-old seedlings were inoculated separately with two different yellow rust isolates that were either avirulent on *Yr6* and *Yr7* {Race 04-44 (WYV 1, 2, 3, 4, 9, CV, Ox/Rob)} or just *Yr6* {Race 03-7 (WYV 1, 2, 3, 4, 7)}. The plants were grown in a cool glasshouse during the early spring and scored for reaction type 3 weeks later using the 0–4 scale proposed by Stakman et al. (1962). The seedling root traits, total root length (TRL) and total root surface area (TRSA) were measured by digital image analysis software of the scanned images of intact root systems of 11-day-old seedlings (two leaf stage) (Bai et al. 2013). Following scanning, the seedling shoot dry weight (SDW) was determined (Bai et al. 2013). Broad-sense heritability values for GY, PH and EM were calculated by the ANOVA tool of software QTL IciMapping version 3.3 (http://www.isbreeding.net/). The broad-sense heritability values for GY, PH and EM were 0.37, 0.46 and 0.77, respectively.

### Linkage map construction and QTL mapping

Function BIN of software QTL IciMapping version 3.3 was used to delete redundant markers and markers with a missing rate higher than 8 % from the 4,021 markers of the Avalon × Cadenza genotype scores (available at http://www.cerealsdb.uk.net). The genetic map was developed using the MAP functionality of QTL IciMapping.

A total of 862 loci, comprising 758 SNPs, 66 SSRs, 22 DArTs and 16 perfect markers, were mapped. The map covered 3,240 cM with an average marker interval of 3.76 cM (Fig. S1).

Multi-environment QTL analysis for GY, yield components, PH, EM and SS was performed by inclusive composite interval mapping (ICIM) (Li et al. 2007) using the MET functionality in the QTL IciMapping software package. In the first step, the probability for entering variables (PIN) was set to 0.0001 and the probability for removing variables (POUT) was set to 0.0002 to select significant markers; the phenotype on marker type model built from the first step of stepwise regression was then used to control the background genetic variation in the second step of QTL interval mapping. A LOD threshold of 3.0 was used to define significant QTL. Also, a LOD threshold at 3.0 was used to identify significant QTL-by-environment effects. Any QTL with phenotypic variance explanation (PVE) higher than 10.0 % was defined as a major QTL. Single-QTL analysis for *Yr*, GY, PH and EM was performed in QTL IciMapping using the BIP functionality. The parameters PIN and POUT were the same as for MET, and also a threshold LOD of 3.0 was used to identify a QTL. Additive effects from single-QTL analyses for GY, PH and EM were used for phenotypic prediction of simulated genotypes in the 4 years 2005–2008.

### QTL used for the UK target genotype

QTL for PH, EM and GY, yield component traits, SS and *Yr* detected in this paper together with favourable *Sbm1* (Bass et al. 2006) and *Vrn*-*A1b* (Yan et al. 2004) alleles were used to design a target genotype. Additionally, four Avalon × Cadenza QTL for TRL, TRSA and SDW (Bai et al. 2013) were included in the model. Intended characteristics of the UK target genotype were: similar PH and EM to Avalon, *Rht*-*D1b* and *Vrn*-*A1b* alleles, high TGW, high GN, long and wide grains, a large root system, resistance to yellow rust and mosaic disease and a maximum GY.

### Simulation experimental design

QU-GENE is a simulation platform for quantitative analysis of genetic models (Podlich and Cooper 1998). Two different models were simulated by QU-GENE. The first model aimed to predict GY, PH and EM performances of new DH lines based on QTL additive effects. In this model, two scenarios were simulated: scenario 1 where QTL were not linked; and scenario 2 in which some QTL linkages (one on 2D for GY and PH, and one on 3A for PH and EM) were present, meaning that QTL had pleiotropic effects. The second model was used to compare the efficiency of three breeding methods to achieve the designed target genotype.

QuLine, an integrated genetic and breeding simulation tool based on the QU-GENE platform, is capable of simulating most breeding methods to develop inbred lines (Wang et al. 2003, 2007a, b; Li et al. 2013). The genotypes of Avalon and Cadenza were used to simulate very large DH populations derived from F_1_ that contained all possible allele combinations of 14 loci for GY, PH and EM. The frequencies of exemplary genotypes were as calculated, nine of them ideal and the other five rare.

**Fig. 1:**
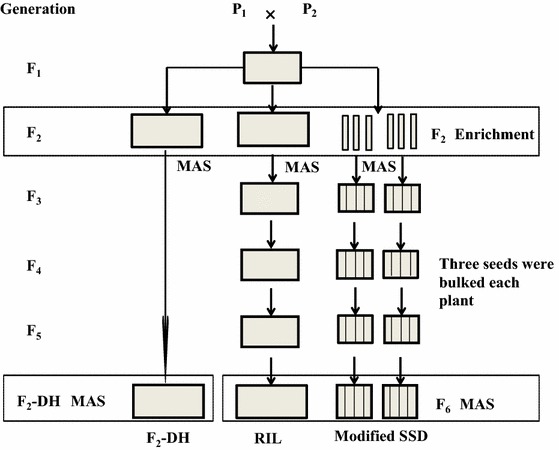
Flow diagrams of the three employed breeding strategies. In the F_2_–DH strategy, DH lines were generated from F_2_. In the modified SSD strategy, three seeds from each plant were harvested and bulked from F_3_ to F_6_ generation. F_2_ enrichment was applied in all three strategies to increase the frequency of target genotypes. In the modified SSD strategy, the pedigree method was used in F_2_ generation. Seeds from selected plants were bulked after F_2_ in the RIL strategy. All seeds were bulked for other generations. The final selection of homozygous target genotype using MAS was conducted in F_2_–DH or in F_6_ generation of the RIL and modified SSD strategy. *Shaded boxes with bold frames* stand for seeds of one generation, either bulked (*larger boxes*) or single harvested by pedigree (*narrow boxes* in modified SSD) or three seeds bulked per plant (*boxes* with subdivisions in modified SSD), *arrows* indicate the production of a new generation. *Frames* indicate generations where MAS is applied

The three simulated breeding strategies were: F_2_–DH, RIL and modified SSD strategy (Fig. 1). In the F_2_–DH strategy, DH lines were generated from F_2_. In the modified SSD strategy, three seeds from each plant were harvested and bulked. F_2_ enrichment by MAS was applied in all three strategies to increase the frequency of target genotypes. In the modified SSD strategy, the pedigree method was used in F_2_ generation. Seeds from selected plants were bulked after F_2_ in the RIL strategy. All seeds were bulked for other generations. The final selection of homozygous target genotype using MAS was conducted in F_2_–DH or in F_6_ generation of the RIL and modified SSD strategy. Each breeding strategy was run 1,000 times. The average number of target genotypes was calculated as a mean of each simulated selection experiment.

### Time required for three breeding strategies

We assumed that it took 1 year to develop DH lines from pollen. For the three strategies, the selected plants were grown in the glasshouse, which allowed growing two generations in 1 year. Under UK field conditions, only one wheat generation is grown per year. Therefore, RIL and modified SSD strategies took 3 years to complete one breeding cycle, and F_2_–DH took 2 years. One breeding cycle was considered in the simulation. A breeding cycle begins with crossing and ends with the generation when selected advanced lines are returned to the crossing block as new parents (Wang et al. 2003). Assuming *F* is the fitness of a population before selection and that TG_l_ and TG_h_ are the genotypic values of the two extreme target genotypes, then the fitness adjusted by target genotypes (*F*_ad_) is

$$F_{\text{ad}} = \frac{{F - {\text{TG}}_{\text{l}} }}{{{\text{TG}}_{\text{h}} - {\text{TG}}_{\text{l}} }} \times 100.$$

The genetic gain per cycle was calculated by the difference on fitness adjusted by target genotype before and after a breeding cycle for GY (Wang et al. 2003). Years in one cycle were used to determine the genetic gain per year. Genetic gains per cycle and per year presented in this study were the means across 1,000 simulation runs.

## Results

### QTL detection

A total of 163 QTL were identified in the Avalon × Cadenza population mainly by multi-environment QTL analysis; among them, 17 QTL were for GY, 47 QTL for yield component traits, 53 QTL for PH, 33 QTL for EM, five QTL for SS and eight QTL for *Yr*.

### GY and yield component traits

A total of 17 QTL for GY were mapped on chromosomes 1A, 1D (two QTL), 2A (two QTL), 2B, 2D (two QTL), 3A (two QTL), 3B (two QTL), 4A, 4B (two QTL), 4D and 5D (Table S1). These QTL explained 1.22–14.53 % of the variation in the individual traits. The QTL *qGY*-*psr*-*2D.1* had the highest additive effect value and PVE. The Cadenza allele had a positive additive effect of 0.37 t/ha on yield. It also showed significant QEI explaining 4.08 % of phenotypic variation. The QTL *qGY*-*psr*-*3B.2* might be another allele of the 3BS GY QTL described by Maccaferri et al. (2008) on 3BS of durum wheat.

For TGW, 11 loci were detected on chromosomes 1D, 2A, 3B, 4B (two QTL), 4D, 5A (two QTL), 5B and 6A (two QTL) (Table S2). These QTL accounted for 1.76–10.78 % of the phenotypic variation with the additive effects in absolute size ranging from 0.63 to 1.72 g. The major QTL was located on 5A with the allele from Cadenza having the largest additive effect. Two QTL on 5A and 6A showed significant QEI, explaining 0.68–2.21 % of the phenotypic variation.

For GN, 11 loci were detected on chromosomes 1A, 1D, 2A, 2D, 3A, 4A, 4D, 5A (two QTL), 6A and 7A, and accounted for phenotypic variation ranging from 2.47 to 11.24 % (Table S3). The additive effects ranged from 400.7 to 856.47 g/m^2^. The QTL detected on 2D contributed the largest additive effect and PVE.

For GRW, nine loci were found on chromosomes 1D, 3D, 4B (two QTL), 4D, 5A (two QTL) and 6A (two QTL) (Table S4). The QTL explained phenotypic variation for GRW ranging from 2.36 to 9.97 %. Two QTL with large additive effect were located on 5A and 6A, with Avalon carrying GRW increasing alleles. Two QTL showed significant QEI, explaining 0.56–2.45 % of phenotypic variation.

For GRL, 16 loci were mapped on chromosomes 2A, 2D, 3A, 3B, 4A, 4B (two QTL), 5A (two QTL), 5B (three QTL), 6A, 6B, 7A and 7D, explaining phenotypic variation from 1.59 to 24.89 % (Table S5). The major QTL on chromosome 5A with the GRL increasing Cadenza allele with an additive effect of 0.15 cm accounted for the maximum percentage of the phenotypic variation for grain length. One QTL on 5B showed a significant QEI with a phenotypic variation of 1.09 %.

### PH and EM

Six QTL for PH on chromosomes 2A, 2D, 3A, 3B, 4D and 5A together explained 67.11 % of the phenotypic variation; among them, three QTL on 2D, 3A and 4D explained over 15 % of the total variation, and PH increasing alleles of these QTL coming from Cadenza having additive effects between 4.42 and 4.92 cm (Table S6). As previously reported, the gibberellin insensitive semi-dwarfing gene *Rht*-*D1* underlies *qPH*-*psr*-*4D*, Avalon carrying the height-reducing allele *Rht*-*D1b* (Griffiths et al. 2012). The QTL on 2D may potentially carry a new allele of *Rh8* (Griffiths et al. 2012). Most QTL for PH were clustered on chromosomes 1B, 2A, 3B, 4B, 5A 5B, 6A and 6B. QTL for PH that showed significant QEI were all located on chromosome 6A.

The major QTL for EM were mapped on 1B, 1D, 3A, 5A and 6A, jointly explaining 31.09 % of the phenotypic variation (Table S7). Most of EM QTL was grouped in clusters of more than three QTL on 1B, 3B, 4A, 5B and 6B. The QTL *qEM*-*psr*-*1D.1* with the largest additive effect, with late EM coming from Avalon, accounted for 9.21 % of the phenotypic variation. A total of eight QTL for EM located on 1D, 3A, 4A, 4D, 6A and 7A showed significant QEI, explaining 0.37–5.28 % of the total variation.

Common QTL for PH and EM were found on chromosomes 3A, 3B and 6A, consistent with previous reports using Meta-QTL analysis (Griffiths et al. 2012). Additionally, five further common QTL for PH and EM, one on 4A, three on 5B and one on 6B were detected. The direction of additive effects for all common QTL was the same except for the 3B QTL, one of the three 5B QTL and the 6A QTL, and these latter loci conferred larger PH and early EM.

### SS

Five QTL for SS were mapped on linkage groups 1B (two QTL), 3B, 5B and 7A, explaining phenotypic variation from 0.85 to 78.27 % (Table S8). Cadenza carried positive alleles for these QTL, except for the 5B QTL. The major QTL on 3B with an additive effect of 20.29 % straw fill contributed 78.27 % to the phenotypic variation for SS. The major QTL detected on chromosome 3B shared a common flanking marker (*BS00071108*) with the 3B PH QTL. The major QTL also showed a significant QEI with a contribution of 1.46 %.

### *Yr*

For yellow rust resistance, four QTL were found for an isolate avirulent on *Yr6* (Race 03-7) located on chromosomes 2B, 2D, 6A and 7B; and four QTL with an isolate avirulent on *Yr7* (Race 04-44), located on chromosomes 2B, 3B, 6A and 7B (Table S9). Individual QTL giving resistance against Race 03-7 explained from 4.59 to 45.68 % of the phenotypic variation with the absolute value of additive effects varying from 0.57 to 1.78, with the major QTL on 7B (*Yr6*), explaining most of the variation. Individual QTL giving resistance against Race 04-44 explained from 5.04 to 51.09 % of the phenotypic variation with the absolute value of additive effects varying from 0.51 to 1.62, with the major QTL on 2B (*Yr7*), explaining most of the variation. The two major QTL alleles increasing yellow rust resistance came from Cadenza. The majority of favourable QTL alleles came from Cadenza except one QTL on 2B for Race 03-7 resistance and one on 3B for Race 04-44 resistance.

### Comparison of the Avalon × Cadenza population and simulated populations

Populations of simulated DH (SDH) lines were created, and three QTL for GY (*qGY*-*psr*-*2D.1*, *qGY*-*psr*-*3A.2* and *qGY*-*psr*-*3B.2*) (Table S1), six QTL for PH (*qPH*-*psr*-*2A.1*, *qPH*-*psr*-*2D*, *qPH*-*psr*-*3A*, *qPH*-*psr*-*3B.1*, *qPH*-*psr*-*4D* and *qPH*-*psr*-*5A.1*) (Table S6), together with five QTL for EM (*qEM*-*psr*-*1B.2*, *qEM*-*psr*-*1D.1*, *qEM*-*psr*-*3A*, *qEM*-*psr*-*5A* and *qEM*-*psr*-*6A.1*) (Table S7) were used to predict phenotypes PH, EM and GY of the simulated individuals. Because of the co-location of QTL on chromosomes 2D and 3A, two scenarios were simulated: scenario 1, all QTL were considered independent; scenario 2, the linkage or pleiotropy of *qPH*-*psr*-*2D* and *qGY*-*psr*-*2D.1*, and *qPH*-*psr*-*3A* and *qEM*-*psr*-*3A* was assumed. The simulations were compared to the performance of the real Avalon × Cadenza population.

**Fig. 2:**
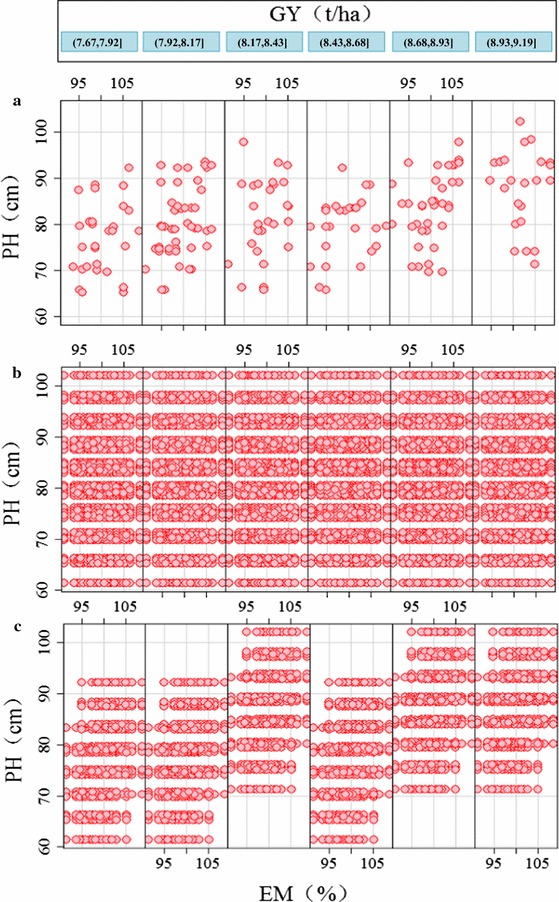
All GY, PH and EM QTL combinations present in the real Avalon × Cadenza population (**a**), scenario 1 (all QTL independent) (**b**) and scenario 2 (some QTL linkages present) (**c**). For Avalon × Cadenza population (**a**), two simulation populations in scenario 1 (**b**) and scenario 2 (**c**), plots of PH versus EM were produced conditional on the variable GY (six GY categories)

In the Avalon × Cadenza population, observed PHs showed a different distribution in different yield categories. The maximum PH increased when GY increased if the first three categories from 7.67 to 8.43 t/ha are regarded (see Fig. 2a). Interestingly, maximum PH was lower in the next yield category of 8.43–8.68 t/ha and increased again as GY increased to maximum values (Fig. 2a). Individuals with near maximum yield were all over 70 cm tall. In a simulated population of 200,000 individuals with no selection, all possible PH, EM and GY combination were found in scenario 1 where there were no linked QTL (Fig. 2b). Many DH lines with very low PH but with the maximum GY were present. The frequencies of these individuals ranged from 0.0025 to 0.009 % (Fig. S2). The frequency of tall individuals with high yield was slightly higher, ranging from 0.0025 to 0.011 % (Fig. S3). For scenario 2, a population of 50,000 sufficed to contain all possible combinations. However, the different height categories did not contain the full phenotypic range as in scenario 1, e.g. short plants were not found in the highest GY category (Fig. 2c). The frequency of tall plants with high yield varied from 0.016 to 0.038 % higher than in scenario 1 (Fig. S4). The population in scenario 2 had a very similar trend to the real population. Using the simulated population from scenario 2 the phenotypic effect of a QTL linkage was revealed by this simplified model. Due to the linkage or pleiotropy of the 2D GY and PH QTL, plants did not achieve the full height range in all the different GY categories. The EM range was also limited because of the linkage or pleiotropy of the 3A PH and EM QTL. For example, no individuals with late EM achieved a height of 70–80 cm; also, individuals with early EM did not grow to a height of over 92 cm (Fig. 2c).

### Predicted performance of a target genotype in simulated populations

For the aim of breeding a wheat variety for UK environment, the following characteristics were assumed for a target genotype: it should carry *Rht*-*D1b*, show a similar PH and EM as Avalon, and carry a combination of all the favourable GY QTL for a high yield. In both simulation scenarios, the standard deviation for PH ranged from 2.18 cm to 4.90 cm, when the phenotype was predicted for the four example environments 2005–2008. The absolute height difference in the 4 years was <10 cm. The standard deviation for EM varied from 1.05 to 5.75 %. All plants were close to the predicted EM phenotype, based on MET QTL, in all 4 years. Nine SDH lines with the ideal genotype from the two scenarios are listed in Table S11 as an example. For scenario 2, SDH2, SDH5 and SDH8 were not found due to the linkage or pleiotropy of the QTL on 3A for PH and EM. The frequency of these nine ideal SDH lines was low and ranged from 0.004 to 0.0096 % in scenario 1, while the frequency for the six lines present in scenario 2 ranged from 0.022 to 0.034 % (Fig. S5). In terms of yield, all these individuals would have performed well in three of the four example years, but not in 2006.

### Target genotype design using identified QTL information

Breeders aim to produce wheat varieties that achieve a high and stable yield. Adaptation to local environments is a prerequisite for a high-yielding variety. However, yield is the result of the performance of many traits, such as plant morphology, flowering time, yield components, root morphology and disease resistance. For resilience to a variety of possibly occurring stressful conditions, an ambitious target genotype would include QTL for traits that confer stress resistance. Using available QTL for the Avalon × Cadenza population, a better target genotype would have favourable alleles for the following traits: apart from GY, PH and EM, large TGW, high GN, long and wide grains, large root system, winter type alleles (particularly *Vrn*-*A1b*) and resistances to mosaic disease and yellow rust. A summary of selected QTL and genes for the ambitious target genotype is shown in Table 1. All selected QTL were major QTL or QTL with large additive effects. The pleiotropic effects or linkage underlying the QTL in the Avalon × Cadenza population were taken into account.

**Table 1:** QTL and genes used in the simulation study and genotypes of DH27, DH61, DH109, DH160, DH182 and the target genotype

QTL locus	DH27	DH61	DH182	DH109	DH160	Target genotype
*qGY*-*psr*-*2D.1* ^a^	qq	QQ	qq	qq	QQ	qq
*qGY*-*psr*-*3A.2*	QQ	qq	QQ	qq	QQ	qq
*qGY*-*psr*-*3B.2*	qq	QQ	QQ	QQ	QQ	QQ
*qTGW*-*psr*-*5A.1*	qq	QQ	qq	QQ	qq	qq
*qGRL*-*psr*-*5A.1*	qq	QQ	qq	qq	qq	qq
*qGRW*-*psr*-*5A.2*	QQ	QQ	QQ	QQ	qq	QQ
*qPH*-*psr*-*2A.1* ^b^	qq	QQ	QQ	qq	QQ	QQ
*qPH*-*psr*-*3A* ^c^	QQ	QQ	qq	QQ	QQ	QQ
*qPH*-*psr*-*3B.1* ^d^	qq	qq	QQ	qq	QQ	qq
*qPH*-*psr*-*4D* ^e^	QQ	QQ	QQ	QQ	QQ	QQ
*qPH*-*psr*-*5A.1*	qq	QQ	qq	QQ	qq	QQ
*qEM*-*psr*-*1B.2*	qq	QQ	qq	qq	qq	qq
*qEM*-*psr*-*1D.1*	QQ	QQ	QQ	QQ	QQ	QQ
*qEM*-*psr*-*5A* ^f^	QQ	qq	QQ	QQ	qq	qq
*qEM*-*psr*-*6A.1* ^g^	qq	QQ	QQ	qq	QQ	QQ
*Vrn*-*A1b* ^h^	qq	QQ	QQ	qq	QQ	QQ
*qTRSA*-*2A* ^i^	qq	QQ	qq	QQ	qq	qq
*qSDW*-*5A* ^i^	qq	QQ	qq	qq	qq	qq
*qTRL*-*5B* ^i^	qq	qq	qq	qq	QQ	qq
*qTRL*-*6A* ^i^	qq	QQ	QQ	qq	QQ	QQ
*Sbm1*-*5D* ^j^	qq	qq	qq	qq	QQ	qq
*Yr6*	qq	QQ	qq	QQ	qq	qq
*Yr7*	qq	qq	QQ	qq	QQ	qq

QQ: allele from Avalon, qq: allele from Cadenza

^a^Common QTL for *qPH*-*psr*-*2D*

^b^Common QTL for *qGY*-*psr*-*2A.1*

^c^Common QTL for *qEM*-*psr*-*3A*

^d^ *qSS*-*psr*-*3B*

^e^Common QTL for *qGN*-*psr*-*4D*

^f^Common QTL for *qGN*-*psr*-*5A* and *qGRL*-*psr*-*5A.2*

^g^Common QTL for *qTGW*-*psr*-*6A.1* and *qGRW*-*psr*-*6A.1*

^h^Yan et al. (2004)

^i^Bai et al. (2013)

^j^Bass et al. (2006)

The following more detailed considerations were made for the target genotype design: to obtain high GY, the favourable genotype should carry the Cadenza allele of the 2D and 3A yield QTL and the Avalon allele of the 3B yield QTL. The 2D Cadenza allele also had a height effect of 4.92-cm increase as linkage or pleiotropy was considered. Moreover, the Avalon allele of *qPH*-*psr*-*2A.1* introduced would increase yield by 0.21 t/ha. Avalon carried the height-reducing *Rht*-*D1b* gene (Griffiths et al. 2012), which was commonly known to also increase GY (Miralles and Slafer 1995; Flintham et al. 1997). In our results, however, *Rht*-*D1b* was linked to GN improvement but not GY. This result could only be explained by a decreasing TGW QTL allele at this locus. The present TGW QTL on 4D was, however, 22 cM apart, which would seem too far away to be the missing locus. Due to low heritability or genetic background effects, the missing QTL was either not detected or appears misplaced and thus could not or not fully be used for the target genotype. The favourable additive effects of 5A and 6A QTL for TGW, GN and GRW were opposed to those of EM at those locations. The Cadenza allele of the 5A QTL for EM was chosen to increase GN, while the Avalon allele of the 6A QTL for EM was chosen to increase TGW and GRW. This selection would shorten the time to EM and thus needed to be counter balanced with other EM QTL. All alleles increasing the root system were selected, and most of them were conferred by Cadenza, except *qTRL*-*6A*. In terms of SS, a filled stem was taken to be advantageous for biomass increase and lodging resistance. Therefore, for QTL on 3B, the positive allele from Cadenza was chosen for the target genotype, resulting in a height reduction of 2.4 cm. To achieve a winter type genotype, which allowed for early autumn drilling favourable under UK conditions, the recessive *Vrn*-*A1b* allele, carried by Avalon was selected. Finally, Cadenza alleles for the mosaic disease resistance loci *Sbm1* on 5D and the major resistance alleles *Yr6* and *Yr7* were selected.

### Parent selection and efficiency of three breeding strategies

To breed the high-yielding target genotype, the initial strategy would be to start with DH lines that carry the three high-yielding QTL alleles. However, given the DH present in the Avalon × Cadenza population, for many of the other selected loci, only the unfavourable alleles would have been available in otherwise suitable parents. Because of that, a different crossing strategy had to be used. Three different initial crosses were tested. For the first cross, DH109 was used as the high-yielding parent and DH160 as the second parent, these parents were chosen according to the complementary allelic state at all selected loci for achieving the target genotype (see Table 1). For the other two crosses (DH61 × DH182 and DH27 × DH61), none of the parents carried all favourable GY alleles, but between the parents all favourable alleles were present. Thus, theoretically, the target genotype should be among the progeny of crosses DH109 × DH160, DH61 × DH182 or DH27 × DH61 if enough progenies were produced; however, there were 16, 13 or 15 target loci still segregating in these crosses, respectively. Direct selection of all homozygous target genotype using molecular markers seemed impractical if realistic population sizes smaller than 10,000 were assumed. The strategy of F_2_ enrichment, aimed at reducing population size and increasing the number of target genotypes (Bonnett et al. 2005; Wang et al. 2007b), was thus applied (Table 2). For cross DH61 × DH182, on average, 9.64 target genotypes were obtained from a population of size 2,000 in the F_2_–DH strategy, using MAS for F_2_ enrichment; 6.22 target genotypes were obtained with the RIL strategy for the same population size when MAS was conducted in F_6_ generation after F_2_ enrichment. For cross DH27 × DH61, 6.45 and 3.82 target lines were achieve with F_2_–DH and RIL strategy, respectively, when a larger population size of 3,000 was used in F_2_, DH and F_6_ generations. When segregating loci were as many as 16, as was the case in cross DH109 × DH160, a population size of 5,000 was needed to generate 7.09 and 4.57 target genotypes for F_2_–DH and RIL strategies, respectively. Only 2.27 target genotypes could be obtained using the modified SSD strategy for cross DH61 × DH182 in spite of the large population sizes (10,000). Moreover, the modified SSD strategy did not produce a single target genotype from crosses DH27 × DH61 and DH109 × DH160.

**Table 2:** Efficiency of breeding strategies and population size of generations

Crosses	Breeding strategies	F_2_ population size before selection and after selection^a^	Population size before final selection (F_2_–DH or F_6_)	Number of target individuals^b^
DH61 × DH182	F_2_–DH	2,000 (47)	2,000	9.64 ± 0.13
	RIL	2,000 (47)	2,000	6.22 ± 0.39
	Modified SSD	10,000 (238)	715	2.27 ± 0.06
DH27 × DH61	F_2_–DH	3,000 (40)	3,000	6.44 ± 0.11
	RIL	3,000 (40)	3,000	3.82 ± 0.35
DH109 × DH160	F_2_–DH	5,000 (50)	5,000	7.09 ± 0.12
	RIL	5,000 (50)	5,000	4.57 ± 0.57

^a^Values in brackets are the population size after F_2_ enrichment

^b^Values are expressed as mean ± SE

**Fig. 3:**
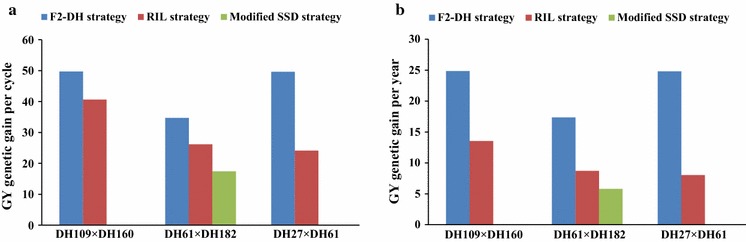
*Bar*-*charts* of GY genetic gain per cycle (**a**) and per year (**b**) calculated for the three breeding strategies for three different crosses (DH109 × DH160, DH61 × DH102 and DH27 × DH61)

In the three analysed crosses, the genetic gains per cycle were always the highest for the F_2_–DH strategy followed by the RIL strategy and, with the lowest values for genetic gains per cycle, the modified SSD strategy (Fig. 3a). Similarly, genetic gains per year were higher for the F_2_–DH strategy than for the other two strategies (Fig. 3b), due to the F_2_–DH method being faster in completing a breeding cycle. Genetic gains per year for cross DH61 × DH182 were higher for the RIL strategy than for the modified SSD strategy, in spite of the breeding cycles being equally long in both strategies (Fig. 3b). Genetic gains per cycle and per year for cross DH109 × DH160 (16 segregating loci) were the highest for both F_2_–DH and RIL strategy, compared with the other crosses. However, the genetic gains advantage of cross DH109 × DH160 over cross DH27 × DH61 (15 segregating loci) was only small using the most effective F_2_–DH strategy. Genetic gains per cycle and per year for cross DH27 × DH61 were higher than for cross DH61 × DH182 (13 segregating loci) using F_2_–DH strategy, while it was the other way round for the RIL strategy.

## Discussion

QTL for GY, yield components, PH, EM, SS and *Yr* were first identified (GY, SS and *Yr*) or re-identified in the Avalon × Cadenza population, most of them as MET QTL and used to suggest an ideal genotype, carrying as many favourable alleles for a UK breeding programme as possible.

### Common QTL for GY with other traits

Previous studies have revealed clusters of QTL for GY, yield components and other yield-related traits. Such clusters were found on chromosomes 1B, 2A, 2D, 3B, 3A, 4A, 4B, 4D, 5A and 6A (Gegas et al. 2010; Zhang et al. 2010; Rustgi et al. 2013). In the Avalon × Cadenza population, one main cluster was found on chromosome 4B with 12 QTL, affecting GY, TGW, GRW, GRL, PH and EM. Other clusters were located on 5A with 12 QTL controlling TGW, GN, GRW, GRL, PH and EM, and 6A with 14 QTL controlling TGW, GN, GRW, GRL, PH, EM and *Yr* (*Yr6* and *Yr7*).

The analysis of common QTL is an important tool to elucidate genetic relationship among traits. In this study, the common QTL between any of the named traits were mapped to chromosomes 1A, 1D, 2A, 2D, 3A, 3B, 4B, 4D, 5A and 6A (Fig. S6). Common QTL for GY and yield component traits were only found for GY and GN on 1A and 2D (Fig. S6). GY increase on 1A and 2D was most likely driven by an increase in GN, as the QTL for GN was found at a very close position with the same flanking markers as the GY QTL on 1A and 2D. No common QTL for GY and TGW was found in this population, although such common QTL are reported in other populations (Simmonds et al. 2014). This could mean that the selection for GN loci in the Avalon × Cadenza population would improve GY by avoiding a trade-off between TGW and GN. The locus for GY on 1D was also associated with the EM QTL with the largest effect, suggesting the allele for late EM would increase yield. Moreover, a linkage or pleiotropy between GY and EM was present on chromosome 3A, again linking late flowering to increased yield at this locus. One common QTL for GY and EM, and one for GN and EM were located on chromosomes 3B and 5A, respectively, linking GY per se or GN increase with earlier EM. Moreover, the two 6A QTL for TGW and GRW also controlled EM, suggesting that the allele for earlier EM could result in a potential GY increase via the yield component GRW. This connection, that GY may be increased by early EM, was in line with the findings of Kuchel et al. (2007). Hence, there was no simple relationship between EM QTL and yield in our trials. For common QTL for EM with GY and GY components, manipulation of those EM loci would lead to GY increase by a total of 0.37 t/ha, GN increase by 589.87 g/m^2^, and TGW increase by 1.62 g. From a breeding perspective, the effect of the chromosome regions associated with early EM on GY and GY components was very intriguing. Earliness had presumably been manipulated either directly or passively by breeders to match the timing of EM in wheat varieties with their target environments (Kuchel et al. 2006). EM primary influence on GY was not mediated through earliness, but rather through their alternative pleiotropic effects on GY (Kuchel et al. 2007).

Three common QTL for GY and PH were mapped on chromosomes 2A, 2D and 3A. The three loci displayed in alleles conferring tallness and increasing yield the same direction of additive effects. Interestingly, the QTL for GN on 4D shared one common marker (BS00107639) with the semi-dwarfing gene *Rht*-*D1b* and both were mapped to a similar location. It is thus likely that *Rht*-*D1b* underlies the 4D QTL for GN. Avalon carried the semi-dwarfing allele *Rht*-*D1b* and also the positive allele for GN. This confirmed that at a given genetic locus, the effect of height increase was not necessarily linked to yield increase, thereby demonstrating that a larger biomass, driven by PH, does not necessarily increase GY via carbohydrate remobilised into the grain. The 5A QTL for GN possibly co-locates with a PH QTL. If this is a common QTL, the height-reducing allele would also confer a higher GN.

Six common QTL for TGW and GRW were found on chromosomes 1D, 4B, 4D and 6A, with QTL clusters on 4B (two QTL) and 6A (two QTL). The positive alleles of these common QTL came from Cadenza also sharing the same effect direction, indicating that at these loci TGW are mainly driven by GRW. This was consistent with the positive correlation coefficient between TGW and GRW (0.82–0.90 in 2007 and 2008). The common QTL on 4B also was found by Gegas et al. (2010) using single-QTL analysis, and further common QTL were detected on chromosomes 2B, 3B, 5A and 6B for the two traits in different wheat populations (Gegas et al. 2010; Ramya et al. 2010). One QTL on 4B (at 30 cM) for TGW and GRW possibly also influenced PH. The Cadenza allele had a positive effect on PH, TGW and GRW, suggesting that height and yield improvement are linked at this locus. No common QTL for TGW, GRW and GRL was found in this study. However, one common QTL for GRL and GN with opposing effects was found on 5A (at 120 cM). Interestingly, Gegas et al. (2010) also found a common QTL on 5A (at 25.1 cM) but for TGW and GRL. This suggests that TGW effects at this locus may be negatively correlated with GN.

The major QTL on 3B for SS was co-located with a PH QTL, and opposing directions of effects meant that the positive allele for SS led to a decrease in crop height. A 3B SS QTL has been reported as associated with sawfly cutting resistance in durum wheat (Houshmand et al. 2003, 2007). Kumar et al. (2007) discovered a SS QTL on 3B, located in close proximity to a yield QTL. However, no association between the major SS locus on 3B and GY was discovered in our study. Another major QTL for SS on 3D (Lanning et al. 2006) did not segregate in the Avalon × Cadenza population. Both loci could be useful for developing new SS genotypes using MAS, but for this study, we only used loci segregating in the Avalon × Cadenza population.

As reported by Li and Niu (2007), *Yr6* was mapped on chromosome 7B, which is rich in rust resistance genes such as *Yr2*, *Lr14* and *Sr17*, and two powdery mildew resistance genes *Pm5* and *Pm47*. *Yr7* was mapped on chromosome 2B, consistent with a previous report by Yao et al. (2006). A number of *Yr* loci, such as *Yr5*, *Yr27*, *Yr31*, *YrV23* and *YrSp* (Luo et al. 2009), were also mapped in this genomic region. The nearest markers for *Yr6* and *Yr7* discovered here, gwm577 and wmc175A, respectively, with distances under 0.4 cM to the respective resistance genes, show potential to be effectively used in MAS to increase the number of resistance loci in a target genotype. However, rust resistance genes may only be of value for a limited duration of time as the pathogens mutate quickly (Hovmoller 2007) and have frequently overcome resistance genes used in commercial wheat varieties. The new appearance of the yellow rust race “Warrior” in 2011 is such a case. Warrior has hit a wide number of commercial wheat varieties such as Kielder, Solstice, Santiago, in spite of them carrying so far functional resistance alleles. Warrior seems to be able to overcome a higher than usual number of resistance genes. A long-term solution for the problem would be the identification of durable rust resistance alleles and their utilisation in wheat breeding programmes. Meanwhile, the duration and efficiency of the rust resistance of a wheat cultivar can be improved by pyramiding multiple race-specific resistance genes (Khan et al. 2005). The effectiveness of this strategy might need to be evaluated in the light of the current “Warrior” race crisis.

### Criterion for designing a target genotype

In this study, a total of 23 loci were used to design an ideal genotype well adapted to UK conditions. The aim was not only a higher yielding genotype under ideal conditions, but to breed for a more resilient genotype, which would still perform well in less favourable years under more stressful conditions, which, to the best of our knowledge, is currently not the case in most breeding programmes. As such, putative “resilient loci” SS, root trait and disease resistance QTL were included in the target genotype. We restricted the employment of loci to QTL identified in the Avalon × Cadenza population.

For QTL or genes with no pleiotropic effects or linkage, such as *qEM*-*psr*-*1B.1*, the decision whether the target genotype should receive the allele from Avalon or from Cadenza is easily made by choosing the allele with the positive effect. Likewise, if favourable alleles of QTL with pleiotropic effects or linkage came from the same parent, they were also easily assigned to the target genotype. This was the case for the common QTL for TGW and GRW, where the allele choice was the same for both traits. However, conflicting trends made the decision which allele to introduce difficult. For example, a common QTL for GN and GRL was located on 5A (at 121 or 122 cM), with the Cadenza allele increasing GN but not GRL. As GN is usually more important for GY improvement than GRL (Peltonen-Sainio et al. 2007), the Cadenza allele should be introduced for an increase in GY. However, if the breeding effort was aimed at end-use quality, a change in grain size might be of interest as milling quality and final flour quality are influenced by grain size (Breseghello and Sorrells 2006). In such a case, it might be favourable to influence grain length and to introduce the Avalon allele. Therefore, decisions on how to deal with trade-offs at common QTL may, to large extent, rely on the aim of a particular wheat breeding programme.

### Breeding resilient cultivars to address the yield gap

Actual yields may vary for a number of reasons such as soil qualities and changing weather patterns, resulting in sub-optimal utilisation of resources. As a consequence, the prediction software revealed that a genotype designed mainly for high yield would have not performed very well in one of the four example environments. It is acknowledged that the frequently observed “yield gap” in farmers’ fields could be addressed through optimising agronomic practices and breeding more resilient cultivars, such as improvement of root traits and SS for drought stress resistance.

### Context-dependency issue in MAS

The lack of consistency of QTL effects across different populations (QTL-by-genetic background) and across environments (QEI) has limited the use of QTL in MAS breeding. In this study, a majority of QTL used for target genotype design were hopefully stable as they were detected by multi-environment QTL analysis and also by single-QTL analysis. All selected PH loci were detected in all 4 years; all EM loci at least in 1 year; the major GY QTL in 4 years; and all yield components and SS loci except GRW were all detected in 2 years. Employing stable QTL could limit the impact of environment context dependency of the marker/trait associations. We used the stable PH and EM QTL to predict the performance of genotypes in four example years and found quite a good accuracy of the prediction. However, for complex traits such as yield, the prediction in one of the example years was not accurate. Even in a single local environment, yield measurements were confounded with many sources of non-genetic variation such as plot size, soil properties and disease pressure. This makes it particularly difficult, time consuming and expensive to identify progenies with maximum yield potential across a sample of environments representative of the target population environments in a given breeding programme. For these reasons, it is highly desirable to identify genetic markers that are diagnostic of yield potential so that superior progenies can be selected via MAS before or during the early stages of field test. In such cases, MAS would still be a useful tool to improve complex traits even if restricted to a specific genetic and environment context (Sebastian et al. 2010).

### Future prospects

The present study was laid out as a proof of principle to investigate the potential of “breeding by design”. Pleiotropic effects of QTL or linkages between QTL, as found in real germplasm, have been taken into account in the genotype design. Results indicated that breeding for a high-performing wheat variety can be done efficiently with a number of potential GY-related loci using genotypic information. The parent selection and selection methods described here could provide a guide for applied marker-assisted wheat breeding. For example, compared with two other simulated crossing schemes, starting with a cross between DH61 and DH182 would result in more target genotypes using a smaller population size which would be of advantage for the breeder. Meanwhile, breeding questions like “how many plants are needed to achieve at least one target genotype” and “which selection method is more advantageous” could be answered by simulation software before the real breeding work is started. In the case of the breeding strategies explored here, and if the cost of DH production is irrelevant, the F_2_–DH strategy gave a clear advantage over the RIL and modified SSD strategy in that a much lower population number was needed to produce target genotypes and more gains per year were obtained. “Breeding by design” with the aid of simulation software provides a cost-effective way to efficiently use a vast amount of genetic data and information available to breeders.

The breeding target presented here was a genotype adapted to a British or very similar environment. The selection of QTL could easily be different if breeding for a different environment, e.g. Southern Europe, was the goal. However, the knowledge of favourable traits and QTL for the adaptation to the chosen environment is, of course, a prerequisite. Genomic selection is an alternative to breeding via MAS and has been successfully applied in maize breeding (Massman et al. 2013). Findings by Poland et al. (2012) indicate that the prediction accuracies are sufficiently high to merit implementation of genomic selection in wheat applied breeding programmes. However, as long as large-scale genotyping is still expensive and limited to expert laboratories, the MAS approach may be the better approach for many breeders. Any advantage of genomic selection over a well-designed MAS strategy as presented here will have to be demonstrated in future applications.

In order to verify the simulation result, the suggested crosses and breeding strategies for the Avalon × Cadenza population will be conducted and compared to the results presented here. This comparison between simulation and real crossing experiments will reveal the accuracy of the simulations. Moreover, the current study focused on a single population in order to use a well-defined example to develop a realistic idea of what is possible. In general, breeding aims to introduce alleles from diverse germplasm into elite lines by MAS to improve GY potential. Similar strategies as laid out here will apply, based on functional polymorphism underlying the QTL described. However, it will be more difficult to estimate effects in different populations due to linkage equilibrium and a lack of diagnostic molecular markers. Hopefully, future simulation studies and breeding efforts will benefit from an increasing availability of validation data and positive outcomes from positional cloning projects in bread wheat for favourable QTL and genes. The development of molecular markers for functional polymorphism is the ultimate goal in successfully applying MAS.

## Electronic supplementary material

Below is the link to the electronic supplementary material.

Supplementary material 1 (PDF 1086 kb)

## Abbreviations

GY: Grain yield
TGW: Thousand grain weight
GN: Grain number
GRW: Grain width
GRL: Grain length
EM: Ear emergence
PH: Plant height
SS: Solid stem
TRL: Total root length
TRSA: Total root surface area
SDW: Shoot dry weight
*Yr*: Yellow rust resistance
QTL: Quantitative trait loci
MAS: Marker-assisted selection
PVE: Phenotypic variance explanation
QEI: QTL-by-environment interaction
