# Supplementary material for: Using the UK reference population Avalon × Cadenza as a platform to compare breeding strategies in elite Western European bread wheat
Source: Mol Breed. 2015 Feb 5;35(2):70. doi: 10.1007/s11032-015-0268-7 (PMC4317512; doi:10.1007/s11032-015-0268-7)

Molecular breeding

# Using the UK reference population Avalon×Cadenza as a platform to compare breeding strategies in elite Western European bread wheat

Juan Ma[1,2], Luzie U.Wingen[2], Simon Orford[2], Paul Fenwick[3], Jiankang Wang[1], Simon Griffiths[2]

Author affiliations:

1. Institute of Crop Science, the National Key Facility for Crop Gene Resources and Genetic Improvement, and CIMMYT China, Chinese Academy of Agricultural Sciences, No. 12 Zhongguancun South Street, 100081 Beijing, China

2. John Innes Centre, Norwich Research Park, Norwich, NR4 7UH, UK

3. Limagrain UK Limited, Rothwell, Market Rasen, Lincolnshire, LN7 6DT, UK

Simon Griffiths (Corresponding author)

Email: simon.griffiths@jic.ac.uk

Table S1 GY QTL detected by multi-environment QTL analysis in the Avalon×Cadenza DH population

| QTL locus | Position | Flanking markers | LOD | PVE (%) | PVE(A) | PVE(AbyE) | Add | AbyE_05 | AbyE_06 | AbyE_07 | AbyE_08 |
|---|---|---|---|---|---|---|---|---|---|---|---|
| *qGY-psr-1A* | 10 | *BS00064205-Tagluten* | 6.77 | 2.74 | 2.72 | 0.02 | -0.19 | 0 | 0.01 | -0.02 | 0.01 |
| *qGY-psr-1D.1* | 2 | *BS00011947-BS00126040* | 3.86 | 1.69 | 1.24 | 0.45 | 0.13 | -0.05 | -0.07 | -0.01 | 0.13 |
| *qGY-psr-1D.2* | 6 | *BS00113456-BS00158980* | 4.28 | 1.76 | 1.27 | 0.49 | 0.13 | -0.07 | -0.06 | 0 | 0.13 |
| *qGY-psr-2A.1* | 40 | *BS00011593-BS00094817* | 8.53 | 3.46 | 3.39 | 0.07 | 0.21 | -0.04 | 0.04 | -0.02 | 0.01 |
| *qGY-psr-2A.2* | 155 | *BS00032383-BS00091763* | 5.49 | 2.05 | 1.98 | 0.07 | 0.16 | 0.04 | 0.01 | -0.03 | -0.02 |
| *qGY-psr-2B* | 184 | *BS00070104-BS00004405* | 3.31 | 1.4 | 1.11 | 0.28 | -0.12 | 0.05 | 0.06 | -0.07 | -0.04 |
| *qGY-psr-2D.1* | 36 | *gwm261-BS00043985* | 35.21* | 14.53 | 10.44 | 4.08 | -0.37 | -0.3 | 0.32 | 0.08 | -0.09 |
| *qGY-psr-2D.2* | 60 | *BS00147940-BS00022210* | 3.84 | 1.95 | 0.77 | 1.17 | -0.1 | 0.07 | -0.21 | 0.07 | 0.08 |
| *qGY-psr-3A.1* | 73 | *cfd79A-BS00022029* | 4.59 | 1.79 | 1.12 | 0.67 | -0.12 | -0.02 | 0.03 | -0.13 | 0.12 |
| *qGY-psr-3A.2* | 87 | *BS00039925-BS00022631* | 8.35 | 3.8 | 2.9 | 0.91 | -0.19 | 0.08 | 0.12 | -0.07 | -0.14 |
| *qGY-psr-3B.1* | 86 | *BS00059416-BS00111311* | 3.4 | 1.28 | 1.17 | 0.12 | 0.12 | 0.04 | 0.02 | 0.01 | -0.07 |
| *qGY-psr-3B.2* | 188 | *BS00100706-BS00003596* | 9.6 | 3.49 | 3.14 | 0.35 | 0.2 | 0.11 | -0.06 | 0 | -0.05 |
| *qGY-psr-4A* | 0 | *BS00010582-BS00064269* | 5.54 | 2.39 | 1.91 | 0.49 | 0.16 | -0.05 | -0.1 | 0.08 | 0.07 |
| *qGY-psr-4B.1* | 16 | *BS00022055-BS00012006* | 3.61 | 1.37 | 1.1 | 0.27 | -0.12 | -0.01 | 0.07 | -0.09 | 0.03 |
| *qGY-psr-4B.2* | 41 | *BS00002637-BS00074787* | 3.02 | 1.22 | 0.38 | 0.84 | -0.07 | -0.06 | 0.18 | -0.07 | -0.06 |
| *qGY-psr-4D* | 0 | *BS99999968-BS00108601* | 3.94 | 1.46 | 1.14 | 0.32 | -0.12 | -0.04 | 0.11 | -0.01 | -0.05 |
| *qGY-psr-5D* | 182 | *BS00067650-BS00179234* | 6.08 | 2.8 | 2.43 | 0.37 | 0.17 | -0.04 | 0.11 | -0.07 | 0 |

PVE: phenotypic variation explained by the QTL; PVE (A): phenotypic variation explained by additive effect; PVE (AbyE): phenotypic variation explained by additive by environment effect; Add: additive effect; AbyE_05: additive effect of QTL in 2005; AbyE_06: additive effect of QTL in 2006; AbyE_07: additive effect of QTL in 2007; AbyE_08: additive effect of QTL in 2008.

Asterisk (*) means QTL by environment effect is significant.

Table S2 TGW QTL detected by multi-environment QTL analysis in the Avalon×Cadenza DH population

| *QTL locus* | Position | Flanking markers | LOD | PVE (%) | PVE(A) | PVE(AbyE) | Add | AbyE_07 | AbyE_08 |
|---|---|---|---|---|---|---|---|---|---|
| *qTGW-psr-1D* | 20 | *BS00130675-BS00022633* | 3.62 | 1.76 | 1.44 | 0.32 | -0.65 | 0.31 | -0.31 |
| *qTGW-psr-2A* | 78 | *BS00052043-BS00011945* | 4.62 | 2.47 | 2.29 | 0.18 | 0.82 | -0.23 | 0.23 |
| *qTGW-psr-3B* | 39 | *BS00037871-wmc326* | 4.15 | 4 | 3.22 | 0.78 | 0.97 | 0.48 | -0.48 |
| *qTGW-psr-4B.1* | 0 | *BS00023204-BS00023051* | 4.31 | 5.18 | 2.88 | 2.3 | -0.92 | -0.82 | 0.82 |
| *qTGW-psr-4B.2* | 30 | *BS00012006-BS00100151* | 6.48 | 3.6 | 3.43 | 0.16 | -1 | 0.22 | -0.22 |
| *qTGW-psr-4D* | 25 | *BS00099053-BS99999977* | 4.53 | 3.69 | 3.45 | 0.24 | -1.01 | -0.27 | 0.27 |
| *qTGW-psr-5A.1* | 5 | *BS00051181-BS00087453* | 17.56* | 10.78 | 10.1 | 0.68 | -1.72 | 0.45 | -0.45 |
| *qTGW-psr-5A.2* | 108 | *BS00041063-BS00105208* | 7.89 | 5 | 4.99 | 0.01 | 1.21 | -0.05 | 0.05 |
| *qTGW-psr-5B* | 160 | *BS00003637-gwm408* | 3.02 | 2.43 | 2.26 | 0.17 | -0.83 | -0.23 | 0.23 |
| *qTGW-psr-6A.1* | 65 | *BS00022947-BS00046964* | 7.42* | 3.55 | 1.34 | 2.21 | 0.63 | -0.81 | 0.81 |
| *qTGW-psr-6A.2* | 80 | *bArc171-BS00011034* | 5.46 | 6.66 | 3.35 | 3.32 | 0.99 | 0.99 | -0.99 |

PVE: phenotypic variation explained by the QTL; PVE (A): phenotypic variation explained by additive effect; PVE (AbyE): phenotypic variation explained by additive by environment effect; Add: additive effect; AbyE_07: additive effect of QTL in 2007; AbyE_08: additive effect of QTL in 2008.

Asterisk (*) means QTL by environment effect is significant.

Table S3 GN QTL detected by multi-environment QTL analysis in the Avalon×Cadenza DH population

| QTL locus | Position | Flanking markers | LOD | PVE (%) | PVE(A) | PVE(AbyE) | Add | AbyE_07 | AbyE_08 |
|---|---|---|---|---|---|---|---|---|---|
| *qGN-psr-1A* | 11 | *BS00064205-Tagluten* | 5.08 | 3.76 | 3.75 | 0.01 | -495.87 | -21.38 | 21.38 |
| *qGN-psr-1D* | 6 | *BS00113456-BS00158980* | 3.6 | 2.99 | 2.91 | 0.08 | 436.74 | 70.92 | -70.92 |
| *qGN-psr-2A* | 153 | *BS00009295-BS00032383* | 3.4 | 2.59 | 2.57 | 0.02 | 411.68 | 36.3 | -36.3 |
| *qGN-psr-2D* | 33 | *gwm261-BS00043985* | 13.63 | 11.24 | 11.11 | 0.13 | -856.47 | 90.85 | -90.85 |
| *qGN-psr-3A* | 95 | *wPt-9215-wmc264* | 7.16 | 5.18 | 4.75 | 0.44 | -558.36 | 169.33 | -169.33 |
| *qGN-psr-4A* | 0 | *BS00010582-BS00064269* | 3.44 | 2.47 | 2.46 | 0.01 | 400.7 | -27.02 | 27.02 |
| *qGN-psr-4D* | 39 | *BS99999977-BS00107639* | 9.66 | 7.97 | 7.96 | 0 | 725.89 | 16.28 | -16.28 |
| *qGN-psr-5A.1* | 12 | *gwm293b-BS00022500* | 3.64 | 2.8 | 2.74 | 0.06 | 422.79 | 62.54 | -62.54 |
| *qGN-psr-5A.2* | 122 | *BS00105208-BS00082218* | 7.32 | 5.57 | 5.33 | 0.24 | -589.87 | 124.62 | -124.62 |
| *qGN-psr-6A* | 87 | *BS00010730-BS00038646* | 4.85 | 3.37 | 2.88 | 0.49 | -435.86 | 178.97 | -178.97 |
| *qGN-psr-7A* | 24 | *BS00073988-BS00023207* | 4.25 | 3.82 | 3.15 | 0.67 | -453.59 | -209.28 | 209.28 |

PVE: phenotypic variation explained by the QTL; PVE (A): phenotypic variation explained by additive effect; PVE (AbyE): phenotypic variation explained by additive by environment effect; Add: additive effect; AbyE_07: additive effect of QTL in 2007; AbyE_08: additive effect of QTL in 2008.

Table S4 GRW QTL detected by multi-environment QTL analysis in the Avalon×Cadenza DH population

| QTL locus | Position | Flanking markers | LOD | PVE (%) | PVE(A) | PVE(AbyE) | Add | AbyE_07 | AbyE_08 |
|---|---|---|---|---|---|---|---|---|---|
| *qGRW-psr-1D* | 20 | *BS00130675-BS00022633* | 4.69 | 2.54 | 2.36 | 0.18 | -0.02 | 0.01 | -0.01 |
| *qGRW-psr-3D* | 63 | *BS00067163-BS00117841* | 3.58 | 2.36 | 2.36 | 0 | -0.02 | 0 | 0 |
| *qGRW-psr-4B.1* | 0 | *BS00023204-BS00023051* | 3.88 | 4.55 | 2.54 | 2.01 | -0.03 | -0.02 | 0.02 |
| *qGRW-psr-4B.2* | 30 | *BS00012006-BS00100151* | 5.82 | 3.15 | 2.98 | 0.17 | -0.03 | 0.01 | -0.01 |
| *qGRW-psr-4D* | 26 | *BS00099053-BS99999977* | 4.5 | 3.76 | 3.42 | 0.34 | -0.03 | -0.01 | 0.01 |
| *qGRW-psr-5A.1* | 17 | *BS00034704-BS00000645* | 7.38* | 3.87 | 3.31 | 0.56 | -0.03 | 0.01 | -0.01 |
| *qGRW-psr-5A.2* | 97 | *BS00051765-BS00071402* | 9.7 | 5.92 | 5.82 | 0.1 | 0.04 | -0.01 | 0.01 |
| *qGRW-psr-6A.1* | 65 | *BS00022947-BS00046964* | 8.64* | 4.25 | 1.79 | 2.45 | 0.02 | -0.03 | 0.03 |
| *qGRW-psr-6A.2* | 80 | *bArc171-BS00011034* | 8.08 | 9.97 | 4.88 | 5.08 | 0.04 | 0.04 | -0.04 |

PVE: phenotypic variation explained by the QTL; PVE (A): phenotypic variation explained by additive effect; PVE (AbyE): phenotypic variation explained by additive by environment effect; Add: additive effect; AbyE_07: additive effect of QTL in 2007; AbyE_08: additive effect of QTL in 2008.

Asterisk (*) means QTL by environment effect is significant.

Table S5 GRL QTL detected by multi-environment QTL analysis in the Avalon×Cadenza DH population

| QTL locus | Position | Flanking marker | LOD | PVE (%) | PVE(A) | PVE(AbyE) | Add | AbyE_07 | AbyE_08 |
|---|---|---|---|---|---|---|---|---|---|
| *qGRL-psr-2A* | 6 | *BS00022024-BS00003735* | 4.71 | 2.07 | 2.04 | 0.03 | 0.04 | 0.01 | -0.01 |
| *qGRL-psr-2D* | 84 | *BS00154619-BS00031475* | 7.06 | 3.11 | 3.1 | 0.01 | 0.05 | 0.00 | 0.00 |
| *qGRL-psr-3A* | 94 | *wPt-9215-wmc264* | 19.64 | 9.07 | 8.97 | 0.11 | 0.09 | -0.01 | 0.01 |
| *qGRL-psr-3B* | 97 | *BS00022512-BS00022025* | 11.45 | 4.8 | 4.71 | 0.09 | 0.06 | -0.01 | 0.01 |
| *qGRL-psr-4A* | 22 | *wPt-7807-BS00106888* | 4.09 | 1.74 | 1.73 | 0.00 | 0.04 | 0.00 | 0.00 |
| *qGRL-psr-4B.1* | 39 | *BS00076033-BS00037020* | 5.21 | 1.88 | 1.27 | 0.60 | -0.03 | 0.02 | -0.02 |
| *qGRL-psr-4B.2* | 59 | *BS00022258-BS00021984* | 4.27 | 2.57 | 1.5 | 1.07 | -0.04 | -0.03 | 0.03 |
| *qGRL-psr-5A.1* | 13 | *gwm293b-BS00022500* | 43.29 | 24.89 | 24.72 | 0.17 | -0.15 | -0.01 | 0.01 |
| *qGRL-psr-5A.2* | 121 | *BS00105208-BS00082218* | 12.85 | 6.66 | 6.59 | 0.07 | 0.08 | 0.01 | -0.01 |
| *qGRL-psr-5B.1* | 109 | *BS00092630-BS00022542* | 4.80* | 1.71 | 0.62 | 1.09 | 0.02 | -0.03 | 0.03 |
| *qGRL-psr-5B.2* | 112 | *BS00049793-bArc74* | 3.95 | 2.38 | 1.19 | 1.19 | 0.03 | 0.03 | -0.03 |
| *qGRL-psr-5B.3* | 160 | *BS00003637-gwm408* | 3.51 | 1.59 | 1.55 | 0.04 | -0.04 | -0.01 | 0.01 |
| *qGRL-psr-6A* | 154 | *BS00011404-cos7Tb* | 8.85 | 3.42 | 2.85 | 0.57 | -0.05 | 0.02 | -0.02 |
| *qGRL-psr-6B* | 55 | *BS00075795-BS00022480* | 3.22 | 1.74 | 1.23 | 0.51 | 0.03 | 0.02 | -0.02 |
| *qGRL-psr-7A* | 3 | *BS00023951-BS00022119* | 5.4 | 2.24 | 2.23 | 0.02 | 0.04 | 0.00 | 0.00 |
| *qGRL-psr-7D* | 94 | *BS00081870-BS00024032* | 4.15 | 1.68 | 1.67 | 0.00 | -0.04 | 0.00 | 0.00 |

PVE: phenotypic variation explained by the QTL; PVE (A): phenotypic variation explained by additive effect; PVE (AbyE): phenotypic variation explained by additive by environment effect; Add: additive effect; AbyE_07: additive effect of QTL in 2007; AbyE_08: additive effect of QTL in 2008.

Asterisk (*) means QTL by environment effect is significant.

Table S6 PH QTL detected by multi-environment QTL analysis in the Avalon×Cadenza DH population

| *QTL name* | Position | Flanking markers | LOD | PVE (%) | PVE(A) | PVE(AbyE) | Add | AbyE_05 | AbyE_06 | AbyE_07 | AbyE_08 |
|---|---|---|---|---|---|---|---|---|---|---|---|
| *qPH-psr-1A.1* | 65 | *BS00003851-BS00080438* | 6.13 | 1.2 | 1.12 | 0.08 | 1.15 | -0.26 | 0.48 | 0.05 | -0.28 |
| *qPH-psr-1A.2* | 69 | *I-10-2W1-BS00022172* | 6.38 | 1.26 | 1.16 | 0.09 | 1.17 | -0.3 | 0.52 | 0.06 | -0.28 |
| *qPH-psr-1B.1* | 24 | *BS00022030-BS00024037* | 3.36 | 0.59 | 0.56 | 0.04 | 0.81 | 0.25 | -0.01 | -0.31 | 0.07 |
| *qPH-psr-1B.2* | 43 | *BS00022648-BS00022324* | 6.28 | 1.09 | 0.98 | 0.11 | 1.08 | 0.28 | -0.08 | -0.57 | 0.37 |
| *qPH-psr-1B.3* | 61 | *BS00095568-BS00076504* | 6.25 | 1.09 | 0.94 | 0.15 | 1.06 | 0.2 | -0.13 | -0.6 | 0.53 |
| *qPH-psr-1B.4* | 76 | *BS00022779-BS00022626* | 5.31 | 0.92 | 0.81 | 0.11 | 0.99 | 0.35 | -0.23 | -0.47 | 0.36 |
| *qPH-psr-1B.5* | 83 | *BS00010436-BS00012029* | 5.58 | 0.97 | 0.84 | 0.12 | 1.01 | 0.3 | -0.43 | -0.32 | 0.46 |
| *qPH-psr-2A.1* | 44 | *BS00094817-BS00070798* | 21.53 | 4.42 | 4.22 | 0.2 | 2.23 | 0.1 | 0.57 | 0.1 | -0.76 |
| *qPH-psr-2A.2* | 121 | *wmc181A-BS00024921* | 3.45 | 0.63 | 0.6 | 0.03 | 0.85 | -0.06 | -0.17 | -0.11 | 0.34 |
| *qPH-psr-2A.3* | 152 | *BS00022150-BS00009295* | 5.35 | 1 | 0.99 | 0.01 | 1.09 | -0.03 | 0.02 | -0.12 | 0.13 |
| *qPH-psr-2B* | 84 | *BS00045163-BS00022493* | 3.43 | 0.68 | 0.65 | 0.02 | 0.88 | -0.16 | -0.01 | 0.27 | -0.1 |
| *qPH-psr-2D* | 37 | *BS00043985-BS00122127* | 82.64 | 20.52 | 20.3 | 0.22 | -4.92 | 0.18 | 0.33 | -0.88 | 0.37 |
| *qPH-psr-3A* | 77 | *BS00022029-bArc19* | 70.98 | 17.04 | 16.8 | 0.24 | -4.45 | 0.21 | 0.63 | -0.84 | 0 |
| *qPH-psr-3B.1* | 9 | *BS00071108-BS00011898* | 25.33 | 5.08 | 4.98 | 0.1 | 2.42 | 0.41 | -0.52 | -0.06 | 0.17 |
| *qPH-psr-3B.2* | 90 | *BS00059416-BS00111311* | 6.75 | 1.31 | 1.28 | 0.03 | 1.23 | 0.01 | 0.12 | -0.31 | 0.18 |
| *qPH-psr-3B.3* | 104 | *BS00060073-BS00110445* | 9.06 | 1.65 | 1.63 | 0.02 | 1.39 | 0.02 | 0.04 | -0.23 | 0.16 |
| *qPH-psr-3B.4* | 110 | *BS00025644-BS00049437* | 8.98 | 1.63 | 1.62 | 0.01 | 1.38 | 0.09 | 0.05 | -0.19 | 0.05 |
| *qPH-psr-3B.5* | 117 | *BS00022219-BS00022051* | 7.47 | 1.38 | 1.37 | 0.01 | 1.27 | 0.01 | 0.11 | -0.22 | 0.1 |
| *qPH-psr-3B.6* | 126 | *BS00024090-BS00003708* | 7.37 | 1.35 | 1.35 | 0 | 1.27 | -0.04 | 0.02 | -0.01 | 0.03 |
| *qPH-psr-3B.7* | 175 | *BS00004108-gwm493* | 3.24 | 0.58 | 0.56 | 0.03 | 0.82 | 0.14 | -0.08 | -0.26 | 0.2 |
| *qPH-psr-3B.8* | 191 | *bArc75-wPt-1081* | 3.91 | 0.71 | 0.65 | 0.06 | 0.88 | 0.16 | 0.05 | -0.45 | 0.24 |
| *qPH-psr-4A.1* | 0 | *BS00010582-BS00064269* | 8.19 | 1.52 | 1.49 | 0.04 | 1.33 | -0.06 | -0.31 | 0.17 | 0.2 |
| *qPH-psr-4A.2* | 28 | *BS99999979-BS00107766* | 6.87 | 1.32 | 1.27 | 0.04 | 1.23 | -0.31 | -0.05 | 0.33 | 0.03 |

| | | | | | | | | | | | |
|---|---|---|---|---|---|---|---|---|---|---|---|
| *qPH-psr-4B.1* | 23 | *BS00022055-BS00012006* | 4.61 | 0.81 | 0.75 | 0.06 | -0.94 | -0.31 | 0.08 | 0.39 | -0.15 |
| *qPH-psr-4B.2* | 70 | *BS00073315-BS00022534* | 5.72 | 1.08 | 1.06 | 0.01 | -1.12 | -0.05 | -0.08 | -0.1 | 0.23 |
| *qPH-psr-4B.3* | 110 | *BS00065800-BS00011038* | 3.73 | 0.68 | 0.67 | 0.01 | -0.89 | -0.12 | -0.06 | 0.19 | -0.01 |
| *qPH-psr-4D* | 48 | *BS00107639-wMAS000002_Rht-D1* | 71.06 | 16.77 | 16.49 | 0.28 | -4.42 | -0.15 | -0.58 | 0.95 | -0.22 |
| *qPH-psr-5A.1* | 5 | *BS00051181-BS00087453* | 16.11 | 3.28 | 3.16 | 0.12 | -1.93 | 0.05 | -0.5 | -0.12 | 0.57 |
| *qPH-psr-5A.2* | 87 | *BS00001736-BS00097996* | 3.11 | 0.6 | 0.47 | 0.13 | 0.75 | 0.14 | 0.03 | 0.46 | -0.62 |
| *qPH-psr-5A.3* | 107 | *BS00041063-BS00105208* | 3.3 | 0.63 | 0.54 | 0.09 | 0.8 | 0.16 | 0.17 | 0.24 | -0.57 |
| *qPH-psr-5A.4* | 150 | *BS00066675-BS00028802* | 8.52 | 1.65 | 1.38 | 0.27 | 1.28 | -0.14 | -0.85 | 0.38 | 0.61 |
| *qPH-psr-5B.1* | 26 | *BS00023008-BS00099534* | 5.28 | 1.03 | 0.93 | 0.1 | 1.05 | -0.16 | 0.58 | -0.13 | -0.29 |
| *qPH-psr-5B.2* | 91 | *BS00081132-BS00034021* | 3.1 | 0.6 | 0.48 | 0.12 | -0.76 | 0.14 | -0.49 | 0.52 | -0.17 |
| *qPH-psr-5B.3* | 100 | *BS00091302-BS00107906* | 3.59 | 0.69 | 0.58 | 0.11 | -0.83 | 0.1 | -0.49 | 0.5 | -0.11 |
| *qPH-psr-5B.4* | 144 | *BS00022408-BS00023216* | 6.25 | 1.2 | 1.17 | 0.04 | -1.19 | 0.15 | -0.35 | 0.15 | 0.05 |
| *qPH-psr-5B.5* | 153 | *BS00068710-BS00024074* | 6.02 | 1.14 | 1.11 | 0.03 | -1.17 | 0.15 | -0.3 | 0.18 | -0.03 |
| *qPH-psr-5B.6* | 160 | *BS00003637-gwm408* | 6.21 | 1.17 | 1.13 | 0.03 | -1.18 | 0.15 | -0.19 | 0.25 | -0.21 |
| *qPH-psr-5B.7* | 163 | *BS00084089-BS00010573* | 6.29 | 1.17 | 1.14 | 0.03 | -1.19 | 0.1 | -0.15 | 0.28 | -0.23 |
| *qPH-psr-5B.8* | 172 | *BS00049213-BS00049403* | 5.2 | 0.94 | 0.88 | 0.06 | -1.04 | -0.14 | -0.1 | 0.47 | -0.23 |
| *qPH-psr-6A.1* | 68 | *BS00054054-BS00105973* | 12.13* | 2.6 | 0.8 | 1.8 | 0.98 | -0.71 | -0.97 | -0.85 | 2.53 |
| *qPH-psr-6A.2* | 75 | *BS00024005-BS00086046* | 10.79* | 1.86 | 0.6 | 1.25 | 0.86 | 2.13 | -0.77 | -0.71 | -0.65 |
| *qPH-psr-6A.3* | 82 | *BS00022553-BS00023092* | 6.46* | 1.55 | 0.4 | 1.16 | 0.69 | -0.64 | -0.83 | 2.03 | -0.56 |
| *qPH-psr-6A.4* | 86 | *BS00002205-BS00047388* | 5.85* | 1.33 | 0.34 | 0.99 | 0.63 | -0.63 | 1.88 | -0.61 | -0.64 |
| *qPH-psr-6B.1* | 3 | *BS00023042-BS00076101* | 4.41 | 0.77 | 0.72 | 0.05 | -0.92 | -0.36 | 0.26 | 0.16 | -0.06 |
| *qPH-psr-6B.2* | 44 | *BS00022081-BS00027771* | 4.82 | 0.88 | 0.86 | 0.02 | -1.01 | -0.13 | -0.13 | 0.22 | 0.04 |
| *qPH-psr-6B.3* | 92 | *BS00087179-BS00046906* | 9.51 | 1.77 | 1.74 | 0.03 | -1.45 | 0 | -0.31 | 0.17 | 0.14 |
| *qPH-psr-6B.4* | 104 | *BS00023972-BS00024084* | 8.26 | 1.56 | 1.52 | 0.04 | -1.35 | 0.06 | -0.37 | 0.19 | 0.11 |
| *qPH-psr-6B.5* | 134 | *wPt-8814-BS00010403* | 4.16 | 0.77 | 0.72 | 0.05 | -0.93 | 0.02 | 0.23 | 0.13 | -0.39 |
| *qPH-psr-6D.1* | 10 | *bArc54-BS00042153* | 5.08 | 0.98 | 0.9 | 0.08 | 1.03 | -0.46 | 0.22 | -0.11 | 0.35 |

| | | | | | | | | | | | |
|---|---|---|---|---|---|---|---|---|---|---|---|
| *qPH-psr-6D.2* | 81 | *BS00022206-BS00168534* | 3.95 | 0.73 | 0.65 | 0.08 | 0.87 | 0.16 | -0.53 | 0.25 | 0.12 |
| *qPH-psr-7B* | 73 | *BS00004376-BS00004403* | 3.37 | 0.65 | 0.63 | 0.02 | 0.86 | 0.01 | -0.01 | 0.24 | -0.23 |
| *qPH-psr-7D.1* | 0 | *BS00122154-BS00132860* | 7.65 | 1.5 | 1.45 | 0.05 | -1.31 | 0.38 | -0.1 | -0.3 | 0.03 |
| *qPH-psr-7D.2* | 22 | *BS00062644-BS00094533* | 3.45 | 0.61 | 0.57 | 0.04 | -0.82 | -0.18 | 0.3 | 0.14 | -0.25 |

PVE: phenotypic variation explained by the QTL; PVE (A): phenotypic variation explained by additive effect; PVE (AbyE): phenotypic variation explained by additive by environment effect; Add: additive effect; AbyE_05: additive effect of QTL in 2005; AbyE_06: additive effect of QTL in 2006; AbyE_07: additive effect of QTL in 2007; AbyE_08: additive effect of QTL in 2008. Asterisk (*) means QTL by environment effect is significant.

Table S7 EM QTL detected by multi-environment QTL analysis in the Avalon×Cadenza DH population

| QTL locus | Position | Flanking markers | LOD | PVE (%) | PVE(A) | PVE(AbyE) | Add | AbyE_05 | AbyE_06 | AbyE_07 | AbyE_08 |
|---|---|---|---|---|---|---|---|---|---|---|---|
| *qEM-psr-1B.1* | 80 | *BS00022530-BS00022392* | 7.66 | 2.21 | 1.61 | 0.6 | 1.2 | -0.41 | 0.04 | 1.16 | -0.79 |
| *qEM-psr-1B.2* | 147 | *BS00109526-BS00107837* | 14.7 | 3.96 | 2.97 | 0.98 | -1.61 | -0.04 | -0.38 | -1.06 | 1.48 |
| *qEM-psr-1B.3* | 162 | *BS00022153-BS00104270* | 5.77 | 1.13 | 0.83 | 0.3 | -0.86 | 0.52 | -0.52 | 0.51 | -0.51 |
| *qEM-psr-1D.1* | 2 | *BS00011947-BS00126040* | 30.24* | 9.21 | 3.93 | 5.28 | 1.87 | 0.49 | -1.93 | 3.34 | -1.9 |
| *qEM-psr-1D.2* | 33 | *wPt-8960-BS00119035* | 3.38 | 0.67 | 0.65 | 0.02 | 0.75 | 0.05 | -0.14 | 0.2 | -0.1 |
| *qEM-psr-2B.1* | 138 | *BS00074661-BS00011187* | 4.55 | 0.95 | 0.72 | 0.23 | 0.79 | 0.34 | 0.54 | -0.54 | -0.33 |
| *qEM-psr-2B.2* | 142 | *BS00004433-BS00011229* | 6.81 | 1.8 | 1.36 | 0.45 | 1.09 | -0.14 | -0.18 | 1.02 | -0.7 |
| *qEM-psr-2D* | 70 | *BS00147940-BS00022210* | 3.67 | 0.83 | 0.79 | 0.03 | 0.84 | -0.1 | 0.13 | 0.21 | -0.23 |
| *qEM-psr-3A* | 77 | *BS00022029-bArc19* | 24.11* | 7.33 | 3.66 | 3.67 | -1.79 | -0.73 | -2.6 | 1.57 | 1.76 |
| *qEM-psr-3B.1* | 84 | *BS00105878-BS00059416* | 5.98 | 1.37 | 1.29 | 0.08 | -1.07 | 0.34 | -0.35 | -0.12 | 0.13 |
| *qEM-psr-3B.2* | 110 | *BS00025644-BS00049437* | 5.72 | 1.31 | 1.24 | 0.07 | -1.04 | 0.28 | -0.35 | -0.07 | 0.15 |
| *qEM-psr-3B.3* | 117 | *BS00022219-BS00022051* | 5.03 | 1.2 | 1.09 | 0.11 | -0.98 | 0.33 | -0.5 | -0.02 | 0.19 |
| *qEM-psr-3D.1* | 2 | *BS00129989-BS00149712* | 4.28 | 0.9 | 0.82 | 0.09 | -0.85 | 0.25 | -0.42 | 0.25 | -0.08 |
| *qEM-psr-3D.2* | 31 | *BS00183559-BS00021920* | 3.34 | 0.8 | 0.73 | 0.07 | -0.8 | 0.2 | -0.42 | 0.05 | 0.17 |
| *qEM-psr-4A.1* | 24 | *BS99999979-BS00107766* | 3.96 | 1.47 | 0.5 | 0.97 | 0.66 | -0.68 | 1.59 | -0.45 | -0.46 |
| *qEM-psr-4A.2* | 38 | *BS00011628-BS00001241* | 9.63* | 2.2 | 1.02 | 1.18 | 0.95 | -1.3 | -0.58 | 1.38 | 0.49 |
| *qEM-psr-4A.3* | 45 | *BS00022125-BS00065607* | 4.42* | 0.72 | 0.31 | 0.41 | 0.52 | 0.98 | 0.02 | -0.5 | -0.5 |
| *qEM-psr-4B* | 56 | *wmc89b-BS00022576* | 3.1 | 0.46 | 0.39 | 0.07 | 0.58 | 0.32 | -0.25 | -0.24 | 0.17 |
| *qEM-psr-4D* | 56 | *BS99999984-BS00023942* | 6.74* | 0.99 | 0.62 | 0.37 | 0.74 | 0.79 | -0.35 | -0.7 | 0.27 |
| *qEM-psr-5A* | 120 | *BS00105208-BS00082218* | 23.83 | 7.4 | 5.78 | 1.62 | 2.25 | -1.22 | 1.59 | 0.71 | -1.08 |
| *qEM-psr-5B.1* | 26 | *BS00023008-BS00099534* | 4.7 | 1.12 | 1.01 | 0.11 | -0.94 | 0.46 | -0.33 | -0.22 | 0.09 |
| *qEM-psr-5B.2* | 127 | *BS00049659-BS00009607* | 4.2 | 0.9 | 0.68 | 0.22 | -0.78 | 0.09 | -0.61 | 0.63 | -0.11 |
| *qEM-psr-5B.3* | 142 | *BS00022408-BS00023216* | 5.24 | 1.03 | 0.85 | 0.18 | -0.87 | 0 | -0.5 | 0.61 | -0.12 |

| | | | | | | | | | | | |
|---|---|---|---|---|---|---|---|---|---|---|---|
| *qEM-psr-5B.4* | 171 | *BS00080474-BS00049213* | 4.49 | 0.99 | 0.76 | 0.23 | -0.83 | 0.11 | -0.67 | 0.61 | -0.05 |
| *qEM-psr-5B.5* | 184 | *BS00049997-BS00028492* | 3.48 | 0.68 | 0.57 | 0.11 | -0.72 | 0 | -0.38 | 0.5 | -0.12 |
| *qEM-psr-5B.6* | 193 | *BS00037023-BS00032082* | 3.34 | 0.79 | 0.67 | 0.12 | -0.78 | 0.22 | -0.55 | 0.27 | 0.06 |
| *qEM-psr-6A.1* | 65 | *BS00022947-BS00046964* | 11.79* | 3.19 | 1.46 | 1.73 | -1.14 | -0.67 | -1.7 | 1.18 | 1.2 |
| *qEM-psr-6A.2* | 79 | *BS00086046-bArc171* | 12.13* | 2.56 | 1.38 | 1.18 | -1.11 | 1.02 | 1.01 | -1.26 | -0.76 |
| *qEM-psr-6B.1* | 53 | *BS00075795-BS00022480* | 4.91 | 1.11 | 1.07 | 0.05 | -0.98 | 0.19 | -0.32 | -0.05 | 0.19 |
| *qEM-psr-6B.2* | 70 | *gwm219-BS00010993* | 7.33 | 1.62 | 1.56 | 0.06 | -1.18 | 0.25 | -0.33 | -0.12 | 0.2 |
| *qEM-psr-6B.3* | 109 | *BS00023972-BS00024084* | 8.59 | 1.84 | 1.77 | 0.06 | -1.26 | 0.16 | -0.24 | -0.21 | 0.3 |
| *qEM-psr-7A.1* | 105 | *BS00022959-BS00023993* | 7.39 | 1.94 | 1.12 | 0.82 | -0.99 | 0.08 | -1.28 | 1.11 | 0.08 |
| *qEM-psr-7A.2* | 120 | *BS00009978-BS00039009* | 13.28* | 3.61 | 2.15 | 1.46 | -1.37 | 0.42 | 1.1 | -1.9 | 0.37 |

PVE: phenotypic variation explained by the QTL; PVE (A): phenotypic variation explained by additive effect; PVE (AbyE): phenotypic variation explained by additive by environment effect; Add: additive effect; AbyE_05: additive effect of QTL in 2005; AbyE_06: additive effect of QTL in 2006; AbyE_07: additive effect of QTL in 2007; AbyE_08: additive effect of QTL in 2008.

Asterisk (*) means QTL by environment effect is significant.

Table S8 SS QTL detected by multi-environment QTL analysis in the Avalon×Cadenza DH population

| QTL locus | Position | Flanking markers | LOD | PVE (%) | PVE(A) | PVE(AbyE) | Add | AbyE_05 | AbyE_06 |
|---|---|---|---|---|---|---|---|---|---|
| *qSS-psr-1B.1* | 0 | *BS00010592-BS00030768* | 3.08 | 0.85 | 0.8 | 0.06 | -2.07 | 0.54 | -0.54 |
| *qSS-psr-1B.2* | 19 | *BS00022030-BS00024037* | 3.83 | 1.04 | 1 | 0.05 | -2.31 | 0.5 | -0.5 |
| *qSS-psr-3B* | 4 | *wPt-4412-BS00071108* | 129.19* | 78.27 | 76.82 | 1.46 | -20.29 | 2.79 | -2.79 |
| *qSS-psr-5B* | 29 | *BS00099534-BS00001525* | 3.5 | 0.93 | 0.92 | 0.01 | 2.22 | -0.24 | 0.24 |
| *qSS-psr-7A* | 142 | *BS00084312-BS00025521* | 3.12 | 0.85 | 0.82 | 0.03 | -2.1 | 0.41 | -0.41 |

PVE: phenotypic variation explained by the QTL; PVE (A): phenotypic variation explained by additive effect; PVE (AbyE): phenotypic variation explained by additive by environment effect; Add: additive effect; AbyE_05: additive effect of QTL in 2005; AbyE_06: additive effect of QTL in 2006.

Asterisk (*) means QTL by environment effect is significant.

Table S9 Yellow rust resistance QTL detected by single QTL analysis in the Avalon×Cadenza DH population

| QTL locus | Position | Flanking markers | LOD | PVE (%) | Add |
|---|---|---|---|---|---|
| *qYrRace 03-7 -psr-2B* | 183 | *BS00070104-BS00004405* | 4.92 | 5.91 | -0.64 |
| *qYrRace 03-7 -psr-2D* | 0 | *bArc124A-BS00128354* | 5.35 | 5.41 | 0.61 |
| *qYrRace 03-7 -psr-6A* | 13 | *BS00067017-gwm334* | 4.08 | 4.59 | 0.57 |
| *Yr6 (7B)* | 58 | *gwm577-BS00010560* | 32.21 | 45.68 | 1.78 |
| *Yr7 (2B)* | 153 | *BS00074789-wmc175A* | 38.3 | 51.09 | 1.62 |
| *qYrRace 04-44 -psr-3B* | 106 | *BS00088281-BS00084911* | 5.65 | 5.04 | -0.51 |
| *qYrRace 04-44 -psr-6A* | 56 | *BS00023976-BS00023086* | 6.95 | 6.24 | 0.57 |
| *qYrRace 04-44 -psr-7B* | 67 | *BS00010181-BS00082001* | 9.14 | 8.45 | 0.66 |

PVE: phenotypic variation explained by the QTL; Add: additive effect.

Table S10 Additive effects of GY, PH and EM MET QTL in 2005 to 2008

| MET QTL | Add by single QTL analysis | | | |
|---|---|---|---|---|
| | 2005 | 2006 | 2007 | 2008 |
| *qGY-psr-2D.1* | -0.67 | -0.35 | -0.33 | -0.46 |
| *qGY-psr-3A.2* | -0.11 | -0.07 | -0.26 | -0.33 |
| *qGY-psr-3B.2* | 0.31 | 0.14 | 0.20 | 0.15 |
| *qPH-psr-2A.1* | 2.61 | 2.83 | 2.39 | 1.80 |
| *qPH-psr-2D* | -5.10 | -5.00 | -5.8 | -4.86 |
| *qPH-psr-3A* | -4.26 | -3.85 | -5.36 | -4.51 |
| *qPH-psr-3B.1* | 2.87 | 1.97 | 2.66 | 2.67 |
| *qPH-psr-4D* | -4.57 | -5.00 | -3.53 | -4.64 |
| *qPH-psr-5A.1* | -1.88 | -2.46 | -2.05 | -1.36 |
| *qEM-psr-1B.2* | -1.68 | -2.05 | -2.7 | -1.38 |
| *qEM-psr-1D.1* | 2.44 | 4.53 | 5.30 | 0.00 |
| *qEM-psr-3A* | -2.52 | -4.42 | -2.93 | -2.11 |
| *qEM-psr-5A* | 1.08 | 4.01 | 3.59 | 1.22 |
| *qEM-psr-6A.1* | -1.83 | -2.87 | 0.04 | 0.06 |

MET: multi-environment QTL analysis; Add: additive effect

Table S11 Predicted performance of ideal simulated DH lines in four years for scenarios 1 (all QTL independent) and 2 (some QTL linkages present)

| Simulated DH lines | Predicted phenotype based on MET QTL | | | Predicted PH (cm) phenotype based on single QTL analysis[a] | | | | Predicted EM (%) phenotype based on single QTL analysis[a] | | | | Predicted GY (t/ha) phenotype based on single QTL analysis[a] | | | |
|---|---|---|---|---|---|---|---|---|---|---|---|---|---|---|---|
| | PH (cm) | EM (%) | GY (t/ha) | 2005 | 2006 | 2007 | 2008 | 2005 | 2006 | 2007 | 2008 | 2005 | 2006 | 2007 | 2008 |
| SDH1 | 75.08 | 98.30 | 9.18 | 71.65 | 78.49 | 78.53 | 71.31 | 98.69 | 95.28 | 101.44 | 97.99 | 9.45 | 7.90 | 9.51 | 10.24 |
| SDH2 | 75.08 | 98.65 | 9.18 | 71.65 | 78.49 | 78.53 | 71.31 | 100.37 | 100.02 | 101.90 | 99.46 | 9.45 | 7.90 | 9.51 | 10.24 |
| SDH3 | 75.08 | 99.07 | 9.18 | 71.65 | 78.49 | 78.53 | 71.31 | 95.98 | 94.23 | 98.02 | 100.42 | 9.45 | 7.90 | 9.51 | 10.24 |
| SDH4 | 75.68 | 98.30 | 9.18 | 73.10 | 79.24 | 79.21 | 72.19 | 98.69 | 95.28 | 101.44 | 97.99 | 9.45 | 7.90 | 9.51 | 10.24 |
| SDH5 | 75.68 | 98.65 | 9.18 | 73.10 | 79.24 | 79.21 | 72.19 | 100.37 | 100.02 | 101.90 | 99.46 | 9.45 | 7.90 | 9.51 | 10.24 |
| SDH6 | 75.68 | 99.07 | 9.18 | 73.10 | 79.24 | 79.21 | 72.19 | 95.98 | 94.23 | 98.02 | 100.42 | 9.45 | 7.90 | 9.51 | 10.24 |
| SDH7 | 76.07 | 98.30 | 9.18 | 73.62 | 77.51 | 79.75 | 73.93 | 98.69 | 95.28 | 101.44 | 97.99 | 9.45 | 7.90 | 9.51 | 10.24 |
| SDH8 | 76.07 | 98.65 | 9.18 | 73.62 | 77.51 | 79.75 | 73.93 | 100.37 | 100.02 | 101.90 | 99.46 | 9.45 | 7.90 | 9.51 | 10.24 |
| SDH9 | 76.07 | 99.07 | 9.18 | 73.62 | 77.51 | 79.75 | 73.93 | 95.98 | 94.23 | 98.02 | 100.42 | 9.45 | 7.90 | 9.51 | 10.24 |

a: additive effect of GY, PH and EM QTL by single QTL analysis see Table S10

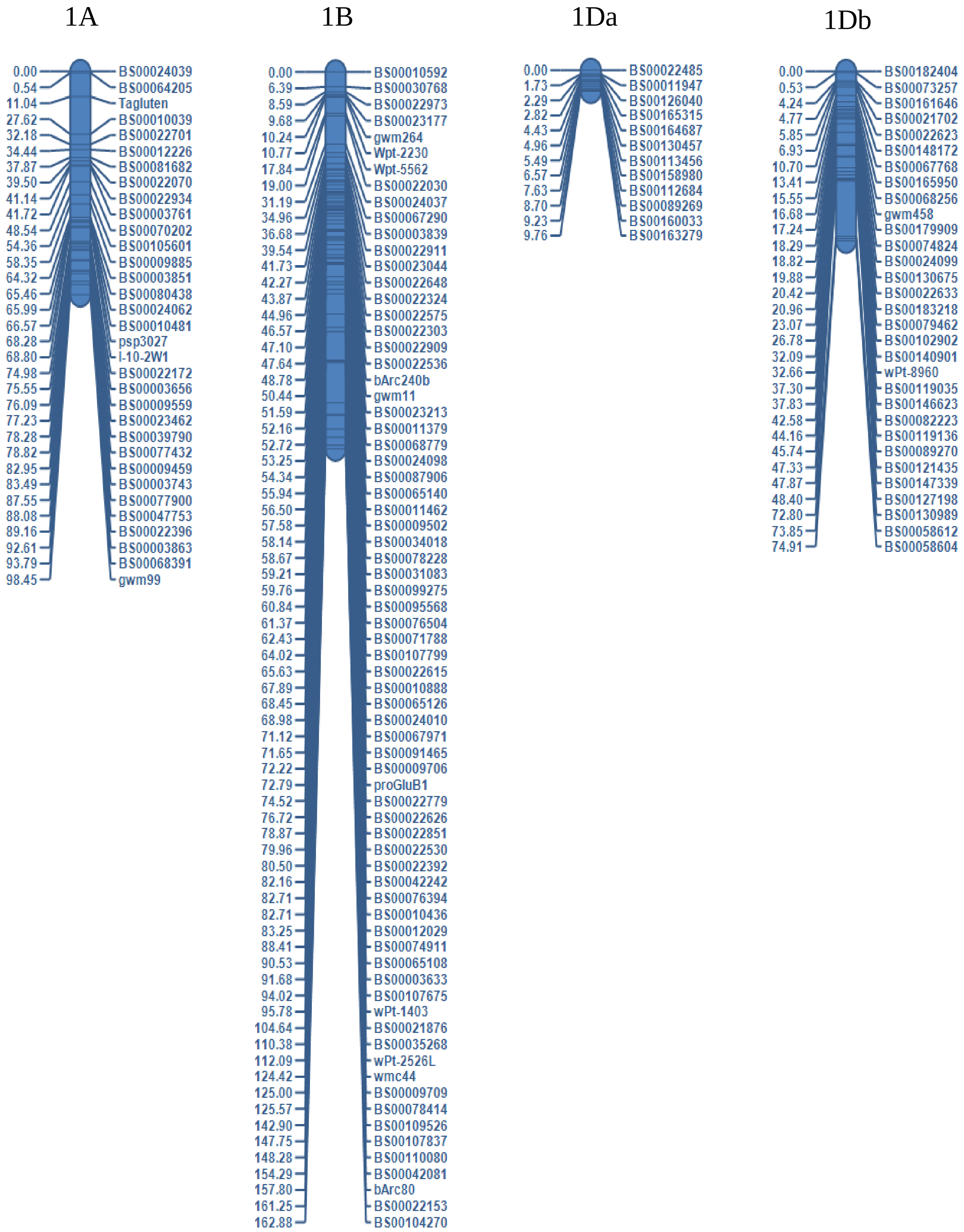


Fig.S1 Genetic map of Avalon×Cadenza population

Fig.S1 continued

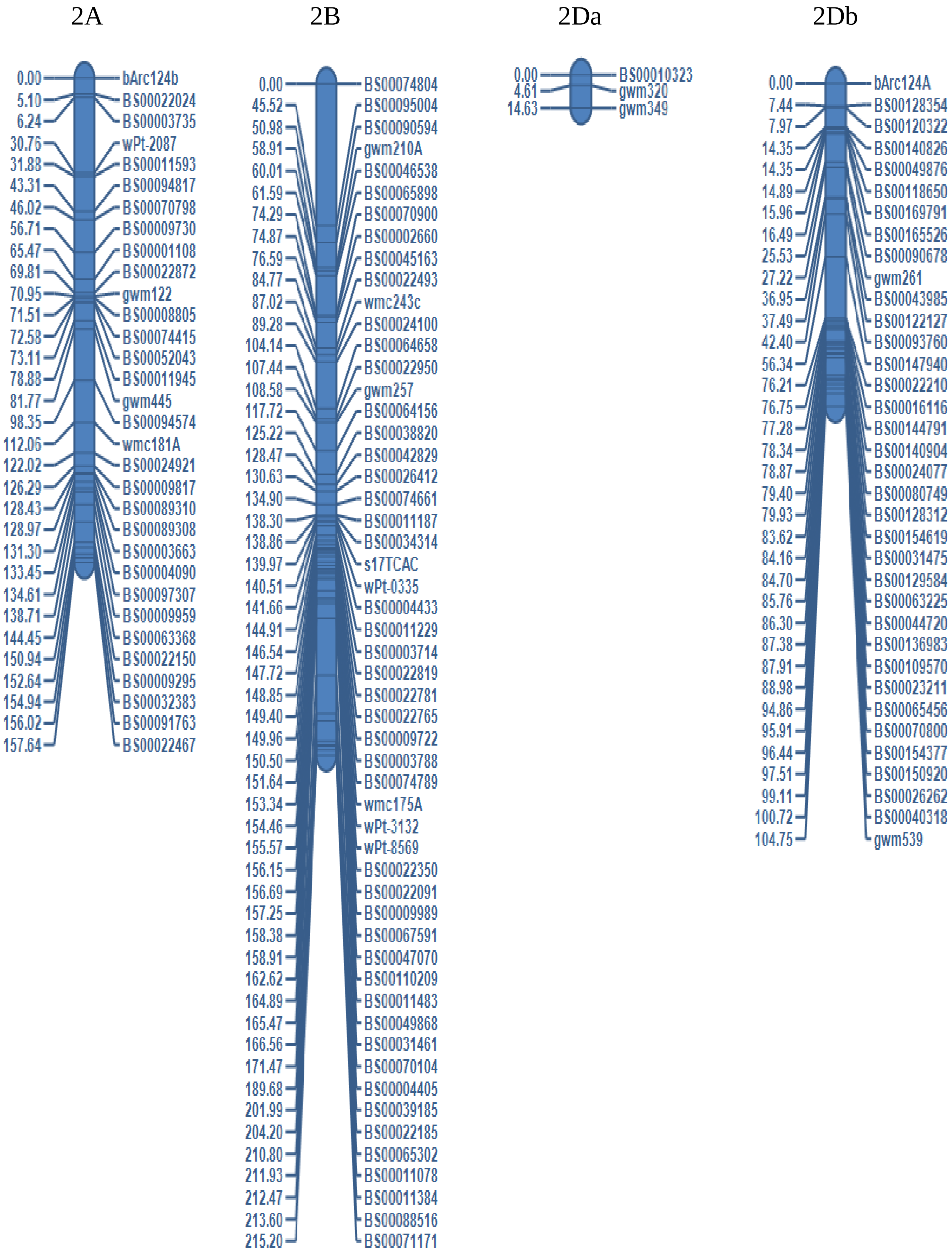

Fig.S1 Continued

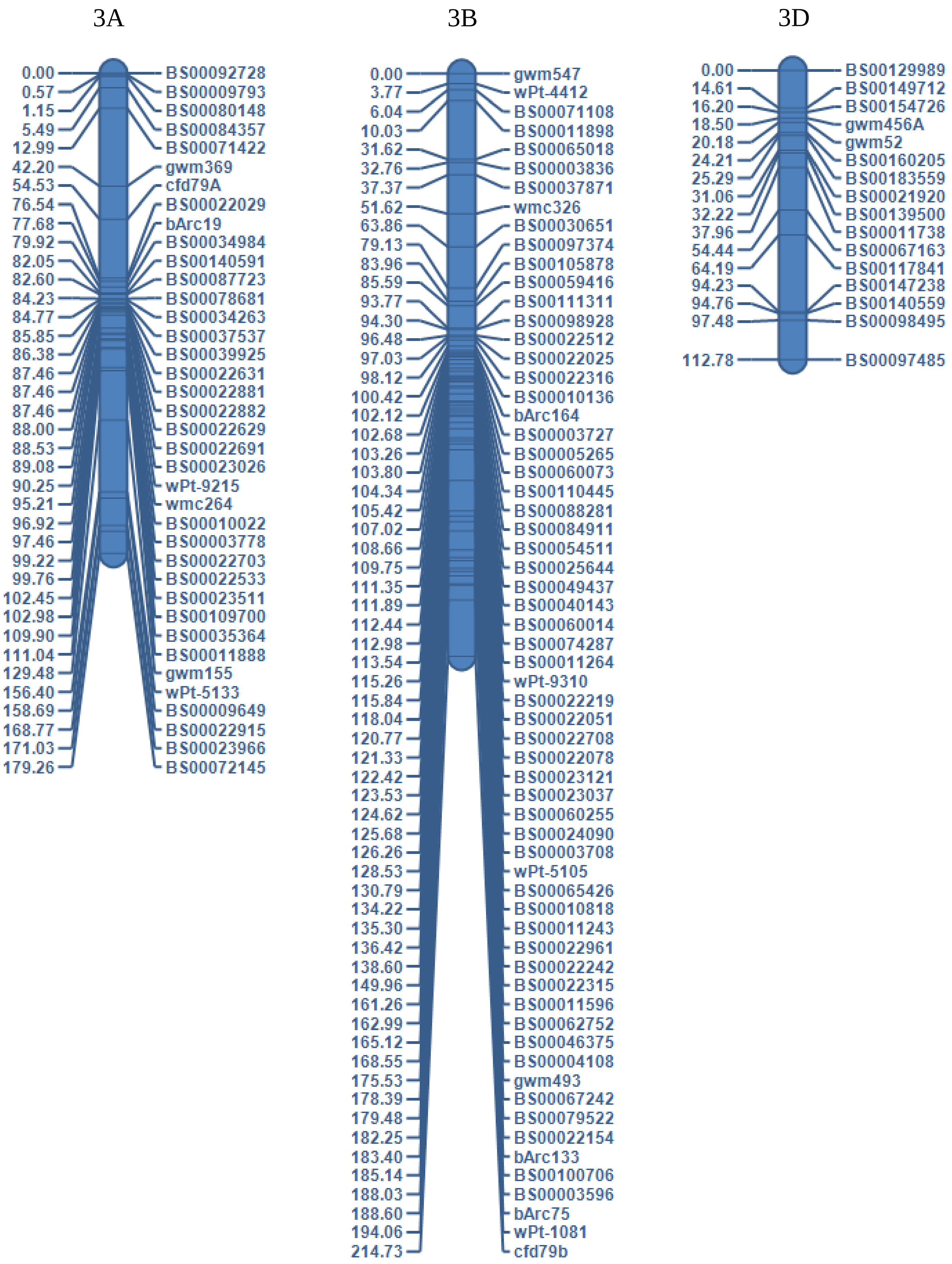

Fig.S1 Continued

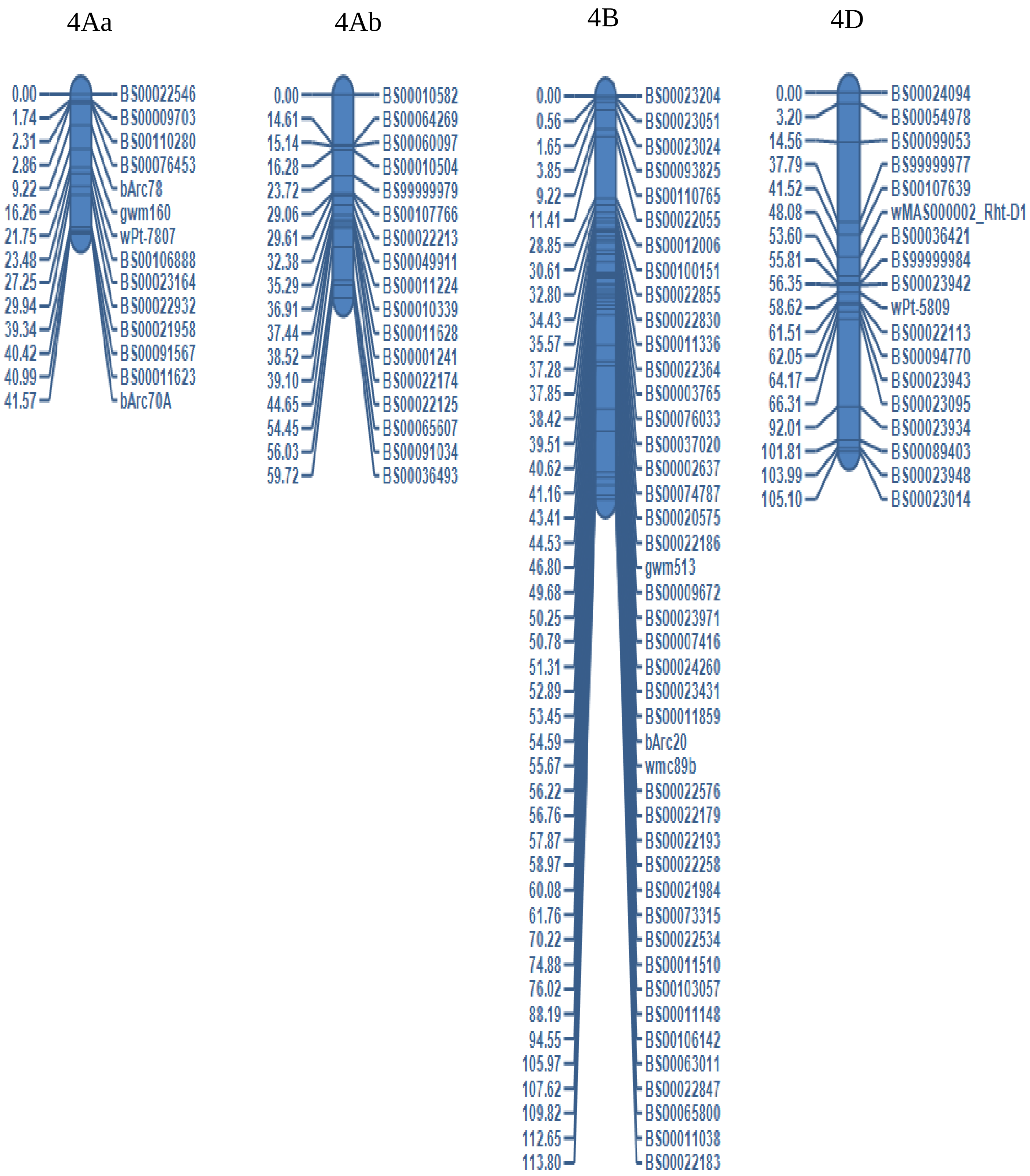

Fig.S1 Continued

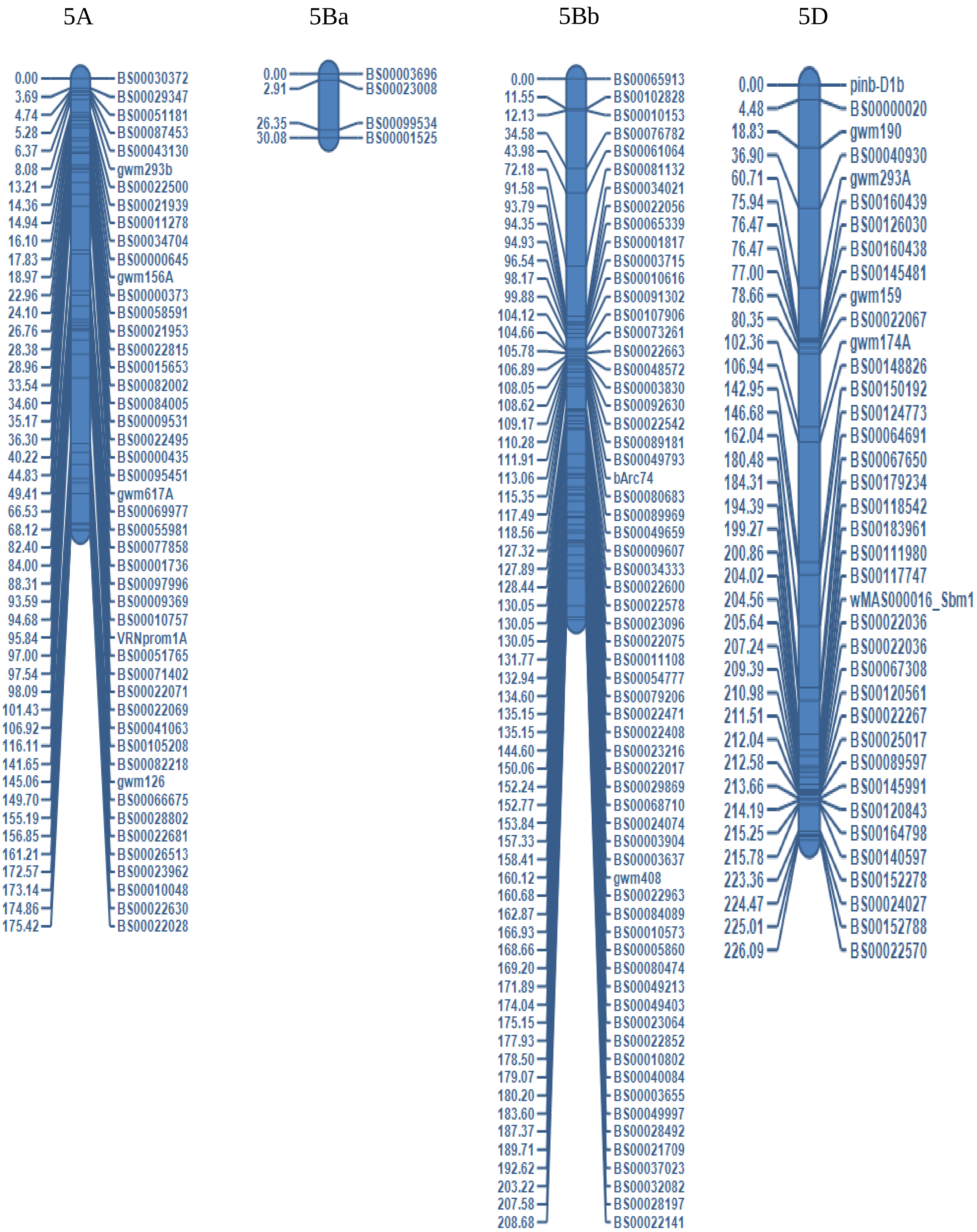

Fig.S1 Continued

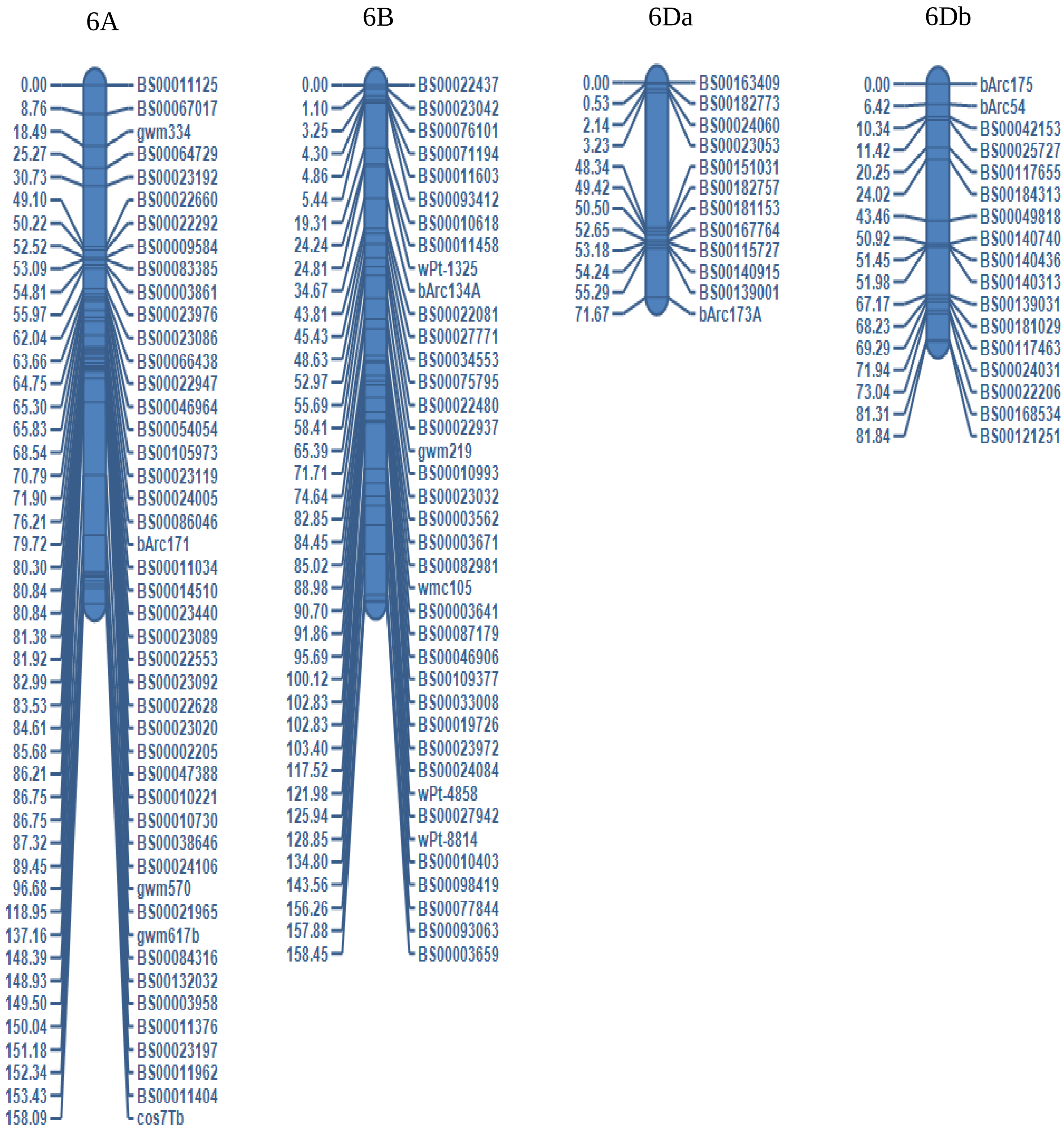

Fig.S1 Continued

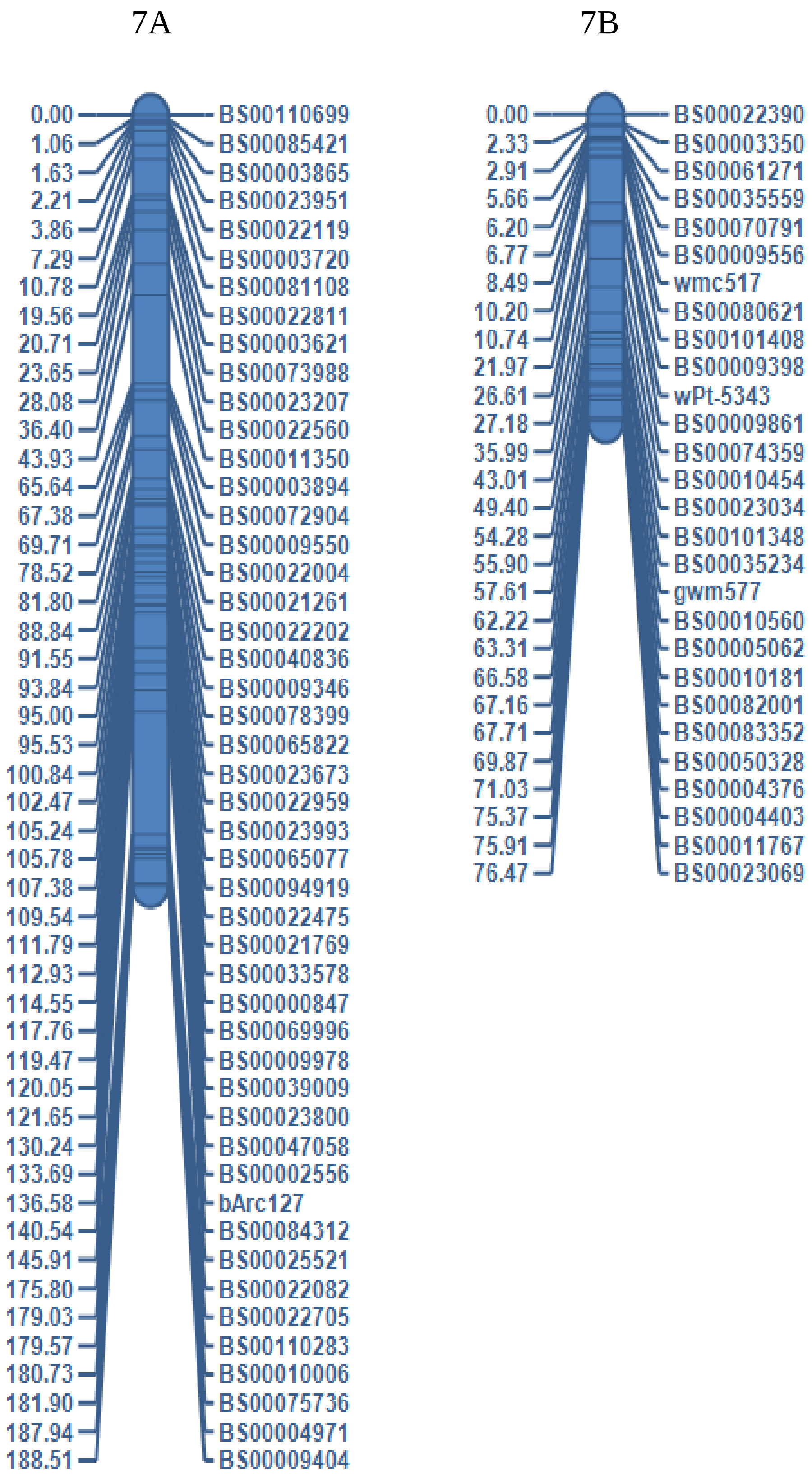

Fig.S1 Continued

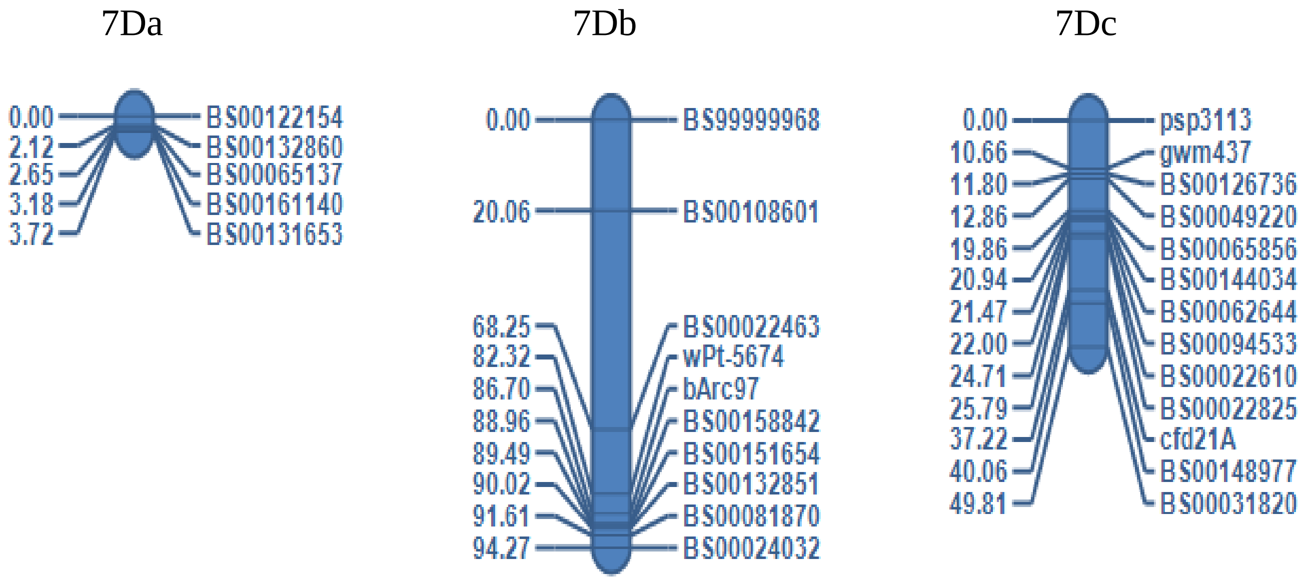

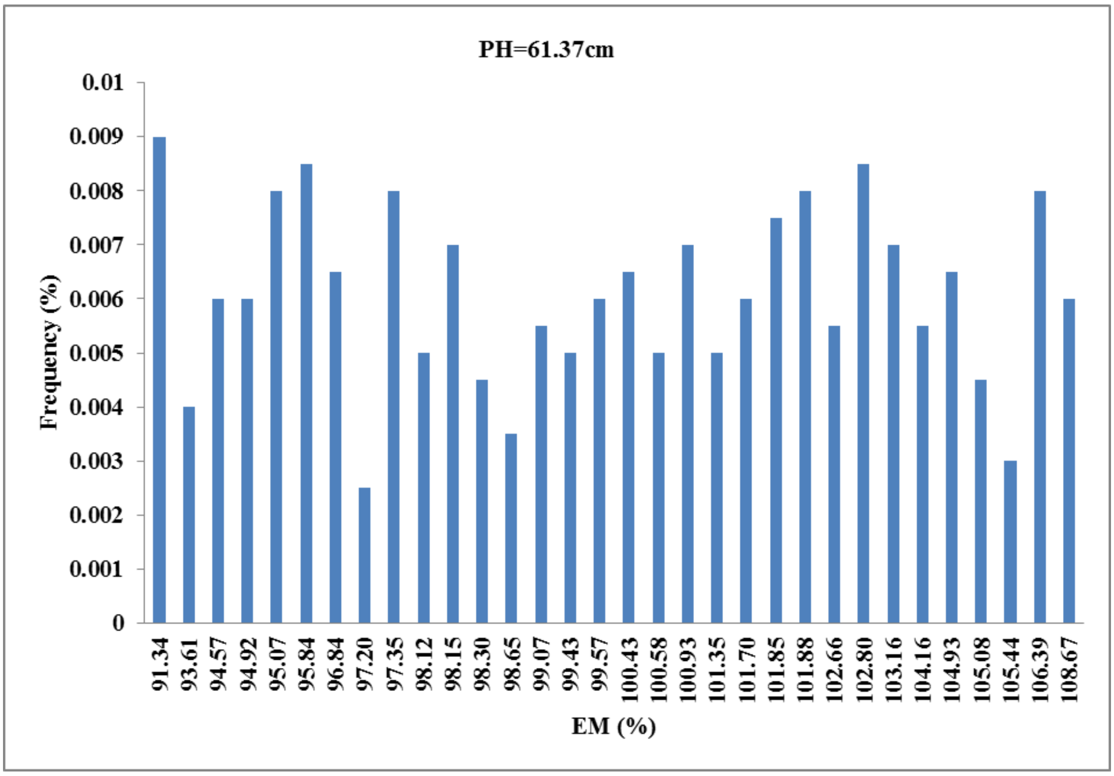


Fig.S2 Frequency of simulated high-yield DH lines with the PH of 61.37cm with different EM in scenario 1

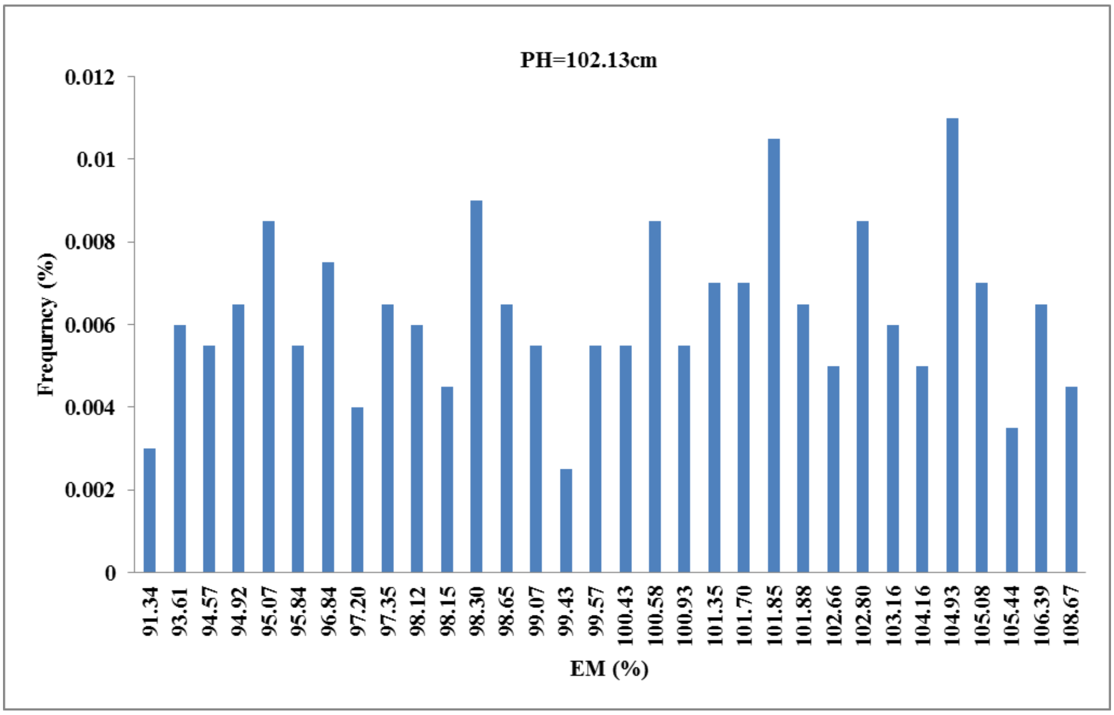


Fig.S3 Frequency of simulated high-yield DH lines with the PH of 102.13cm with different EM in scenario 1

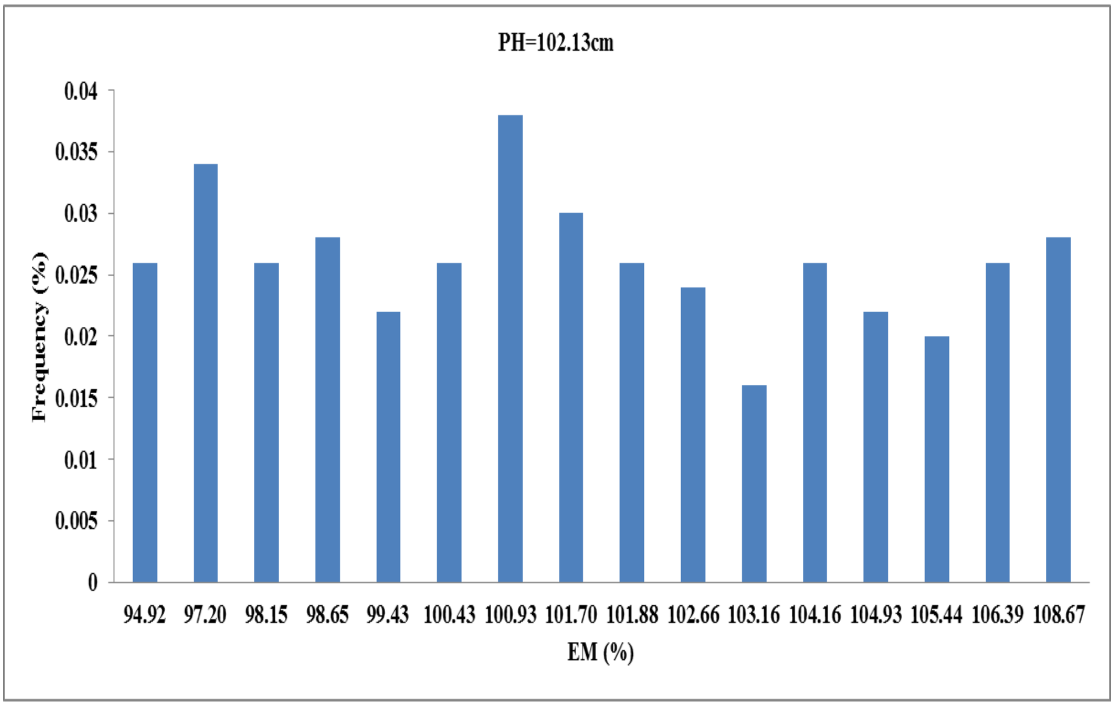


Fig.S4 Frequency of simulated high-yield DH lines with the PH of 102.13cm and different EM in scenario 2

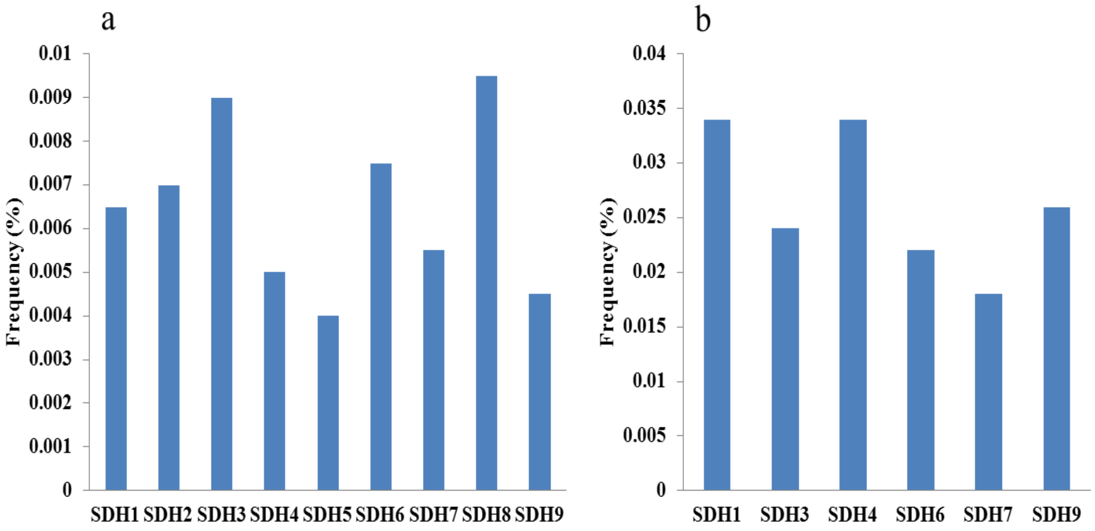


Fig.S5 Frequency of ideal simulated DH lines for scenario 1 (a) and scenario 2 (b)

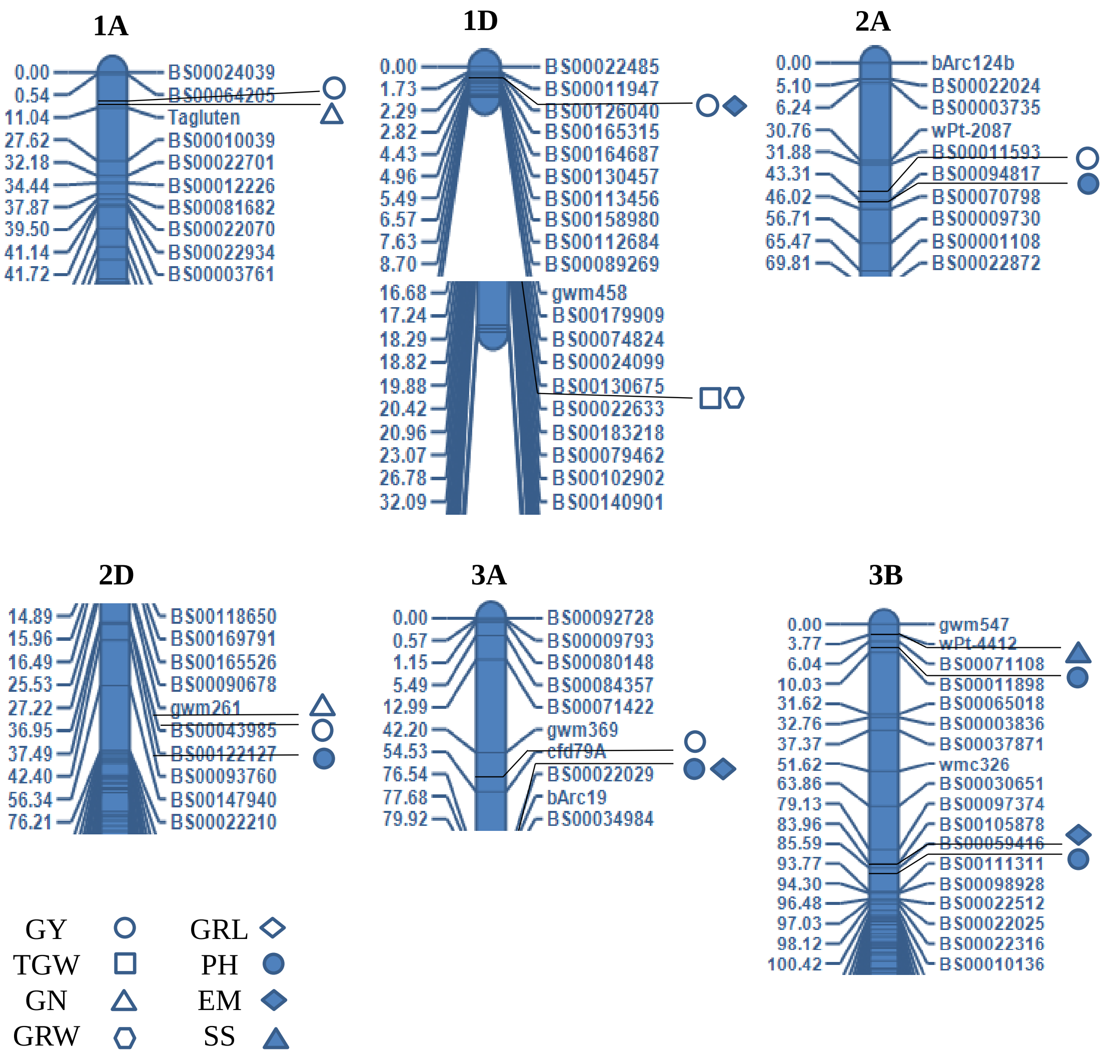


Fig.S6 Pictures of map locations of common QTL for GY (empty circle), yield components (TGW=square, GN= triangle, GRW=hexagon, GRL=diamond, all empty), PH (filled circle), EM (filled diamond) and SS (filled triangle).

Fig.S6 Continued

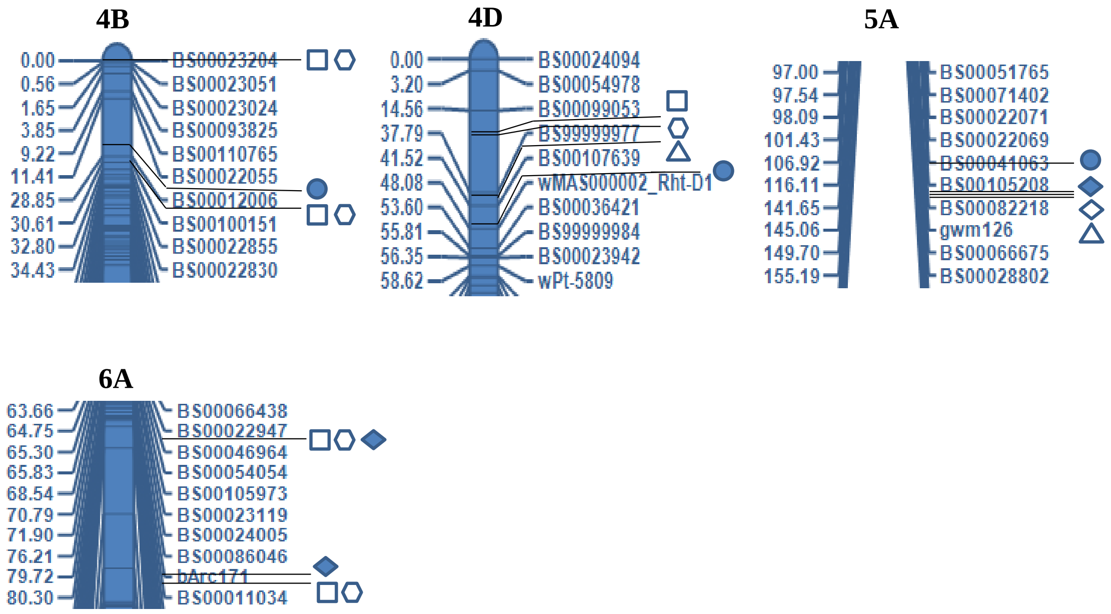

Supplement: Supplementary file 1 — Supplementary material 1 (PDF 1086 kb) [file 11032_2015_268_MOESM1_ESM.pdf]
